# The transcriptional landscape of glycosylation-related genes in cancer

**DOI:** 10.1016/j.isci.2024.109037

**Published:** 2024-01-29

**Authors:** Ernesto Rodriguez, Dimitri V. Lindijer, Sandra J. van Vliet, Juan J. Garcia Vallejo, Yvette van Kooyk

**Affiliations:** 1 Amsterdam UMC Location Vrije Universiteit Amsterdam, Molecular Cell Biology and Immunology, De Boelelaan 1117, Amsterdam, the Netherlands; 2 Cancer Center Amsterdam, Cancer Biology and Immunology, Amsterdam, the Netherlands; 3 Amsterdam Institute for Immunology and Infectious Diseases, Cancer Immunology, Amsterdam, the Netherlands

**Keywords:** Biochemistry, Cancer systems biology, Transcriptomics

## Abstract

Changes in glycosylation patterns have been associated with malignant transformation and clinical outcomes in several cancer types, prompting ongoing research into the mechanisms involved and potential clinical applications. In this study, we performed an extensive transcriptomic analysis of glycosylation-related genes and pathways, using publicly available bulk and single cell transcriptomic datasets from tumor samples and cancer cell lines. We identified genes and pathways strongly associated with different tumor types, which may represent novel diagnostic biomarkers. By using single cell RNA-seq data, we characterized the contribution of different cell types to the overall tumor glycosylation. Transcriptomic analysis of cancer cell lines revealed that they present a simplified landscape of genes compared to tissue. Lastly, we describe the association of different genes and pathways with the clinical outcome of patients. These results can serve as a resource for future research aimed to unravel the role of the glyco-code in cancer.

## Highlights

• Deep analysis of the expression of glycosylation-related genes and pathways cancer
• scRNA-Seq data allowed us to resolve the expression of genes at a single cell level
• Cancer cell lines present a simplified landscape of genes compared to tumor tissue
• Different genes and pathways are associated with the survival of patients

## Graphical abstract

**Figure:**
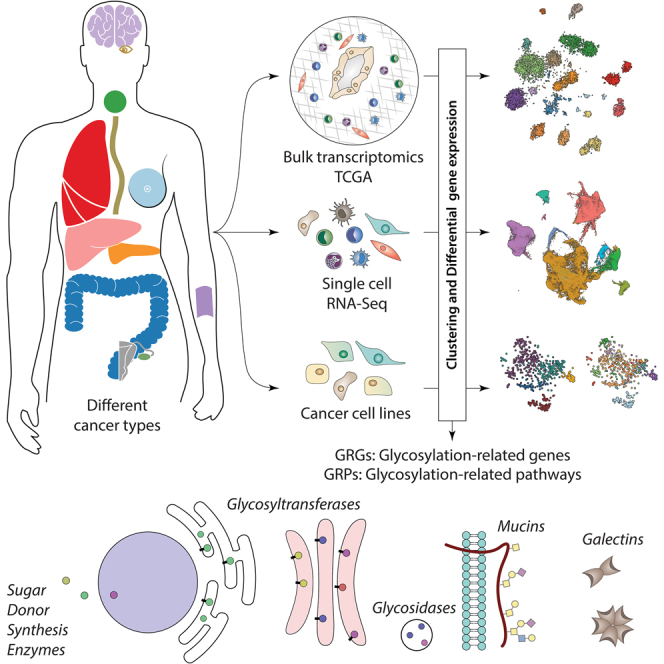

## Introduction

Glycosylation is a complex and highly regulated metabolic pathway that leads to the enzymatic synthesis of glycan structures attached to different macromolecules, such as proteins, lipids, or RNA, collectively known as glyco-conjugates.^1^^,^^2^^,^^3^ In the human genome, hundreds of genes encode for proteins that directly or indirectly contribute to the diverse array of glycan structures found in the cell, collectively known as the *glycome*.^4^ Alterations in the glycome are a universal feature of malignant transformation and different cancer-associated glycans have been linked with tumor progression, by playing key roles in cell-to-cell adhesion, modulation of signaling receptors, metastasis and induction of the tolerogenic microenvironment.^2^^,^^3^^,^^5^ Moreover, several biomarkers used in the clinic for diagnostic or prognostic purposes are glycan structures or glycoproteins, as is the case for CA19-9, a carbohydrate structure also known as sialyl Lewis a (sLe^a^), which serum levels are increased in gastrointestinal cancers.^2^^,^^6^

One of the most studied glycoproteins in cancer are the mucins, proteins containing hundreds of glycosylation sites in their backbone. In normal tissue, mucins are expressed in the apical membrane of polarized epithelial cells; however, cancer cells often lose their polarization, resulting in the presence of mucins in the tumor microenvironment and the blood stream.^5^ In fact, several mucins are currently used as biomarkers in the clinic, as MUC1 (CA15-3) in breast cancer and MUC16 (CA125) in ovarian cancer.^2^^,^^7^^,^^8^

Additionally, glycan structures can be recognized by carbohydrate binding proteins, also called lectins. In humans, almost two hundred genes encode for lectins that differ in their glycan specificity and cellular distribution.^3^^,^^4^ Galectins are a family of soluble lectins with specificity toward glyco-conjugates containing LacNac moieties (Galβ1-4GlcNAc) that can be secreted by tumor and stromal cells, functioning as autocrine or paracrine signals that work in a glycan-dependent manner.^9^ They have been functionally involved in a broad range of biological processes in cancer, as RNA splicing, anti-tumor immune response and cell migration and differentiation, among others.^9^

In the last decade, the extensive use of transcriptomic analysis in cancer research has revolutionized our current understanding of the disease, generating an important amount of publicly available datasets.^10^^,^^11^ Despite that the role of glycosylation has been widely overlooked, some previous reports have shown the association of glycosylation-related genes with different tumor types or the clinical outcome of patients with specific malignancies.^12^^,^^13^^,^^14^^,^^15^ Here, we characterized the expression of glycosylation-related genes (GRGs) and pathways (GRPs) in a pan-cancer setting using bulk and single cell transcriptomic datasets from tumor tissues and cell lines. This allowed us to identify GRGs and GRPs associated with different tumor types, characterize the differential contribution of cancer and stromal cells to the overall tumor glyco-code and study their association with the clinical outcome of patients.

## Results

### Identification of cancer-specific glycosylation signatures

**Figure 1:**
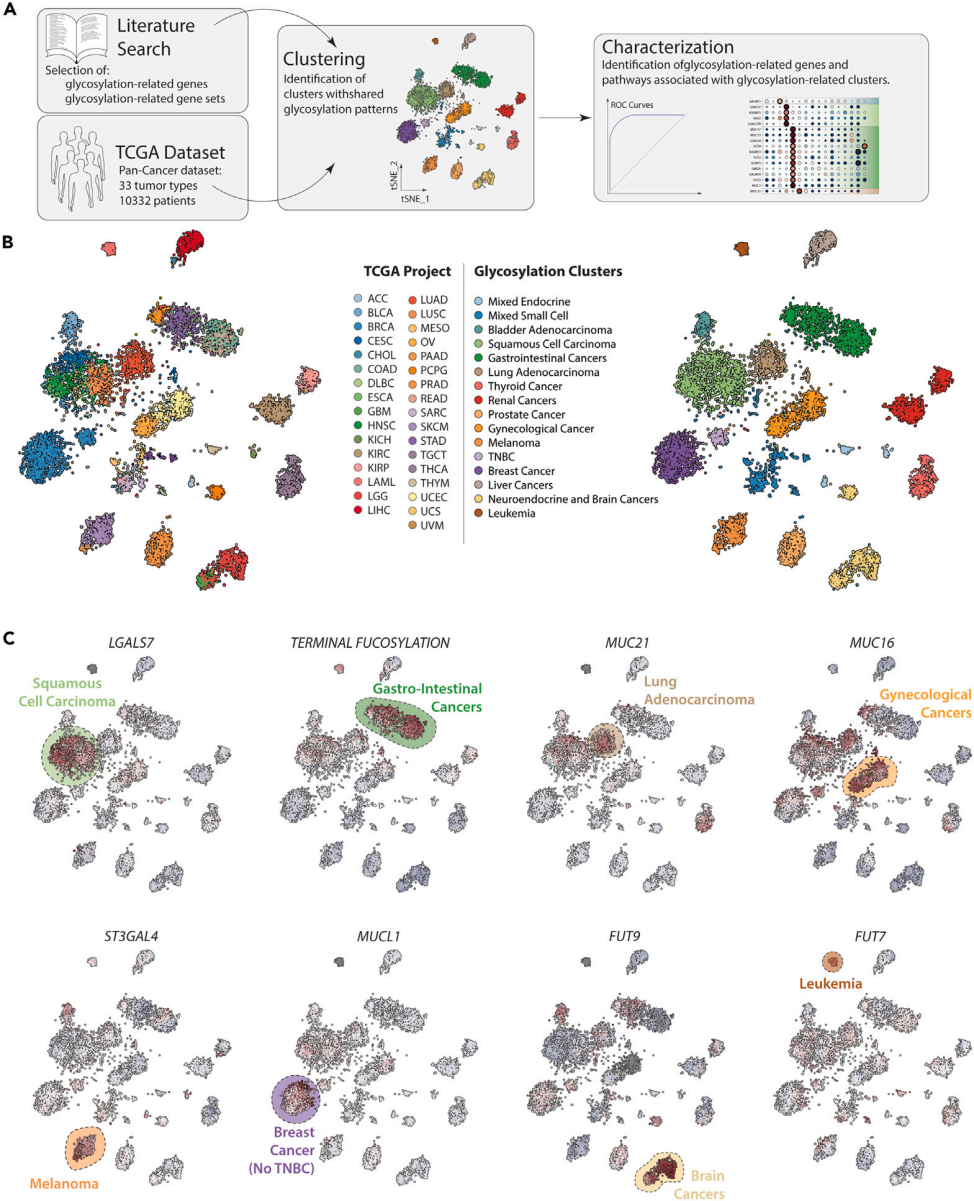
Identification of specific glycosylation signatures of tumor (A) Workflow used in this article for the transcriptomic analysis of glycosylation-related genes and pathways in cancer. (B) TSNE plot showing TCGA projects (left) and glycosylation-clusters (rights), obtained after consensus clustering using GRGs. (C) Expression of selected specific GRGs or GRPs in the different glycosylation clusters.

To perform a transcriptomic analysis of cancer focused in glycosylation, we started by selecting a set of genes involved in glycosylation (glycosylation-related genes or GRGs). For this, we selected genes encoding for *enzymes* involved in the synthesis or degradation of glyco-conjugates and activated sugar donors, as well as the ones corresponding to glycan-specific membrane *transporters*, based on previous literature^4^^,^^12^^,^^15^ (Figure 1A; Table S1). We also included genes that encode for the protein backbone of *mucins*, carriers of tumor associated glycan structures that can be present in circulation; as well as *galectins*, as they can function as glycan-dependent signaling molecules in the tumor microenvironment and can be associated with diverse phenotypes of cancer cells, as we showed previously^13^ (Table S1). Moreover, we made use of gene sets (both custom-made and from different databases) for the transcriptomic analysis of glycosylation-related pathways (GRPs) using gene set enrichment analysis (Table S2).^16^^,^^17^^,^^18^^,^^19^

Firstly, we explored whether we could identify GRGs and GRPs to be associated with different cancer types. For this, we used the TCGA pan cancer dataset which contains bulk RNA-Seq data from 10322 samples derived from 33 different tissues (Table S3). Given that transcriptomic signatures can be shared between samples from different tumor types, we started our analysis by performing consensus clustering using the glycosylation-related genes. This analysis led to the identification of 16 clusters of tumor samples with similar GRG expression (Figure 1B). In line with previous analysis, the tissue of origin heavily influences the clustering, with samples of a given tissue generally grouped together in the same glycosylation cluster, with the exception of a series of heterogeneous cancers, such as breast (BRCA), bladder (BLCA), cervical (CESC), and esophagus (ESCA) cancers (Figure S1).^10^ This is also reflected in the clusters that present only samples from the same organ or tissue type, as is the case for thyroid (THCA), renal (renal clear cell [KIRC] and papillary cell [KIRP] carcinomas), prostate (PRAD), and blood (acute myeloid leukemia [LAML]) cancers (Figures 1B and S1). This suggests that the main determinant of GRG pattern in tumors is dictated by the tissue in which the tumor originates.

We also observed samples that are grouped based on morphological features or tissue/organ similarity: S*quamous Cell Carcinomas* (SCC; light green), are grouped together independent of the tissue in question (Lung, Head and neck, Cervix, Esophagus or Bladder); the same for *Gastro-Intestinal cancers* (colon [COAD], rectal [READ], stomach [STAD] and pancreatic [PAAD] cancers; green); *Gynecological cancers* (ovarian [OV] and endometrial [UCEC] cancers; orange); *Melanomas* (skin cutaneous [SKCM] and uveal [UVM], dark orange), derived from both the eye and the skin; *Liver cancers*, including cholangiocarcinomas (LIHC and CHOL, respectively; gray); *Neuroendocrine and Brain Cancers* (pheochromocytoma and paraganglioma [PCPG], low grade gliomas [LGG] and glioblastoma [GBM]; yellow). Interestingly, we also observed that samples of patients with *triple-negative* breast cancer (TNBC; light purple) group together to form an independent cluster from the rest of breast cancer subtypes (Figure 1B). We also observed two mixed clusters, which we denominate as *endocrine* (adrenocortical carcinoma [ACC], chromophobe renal cell carcinoma [KICH] and thymoma [THYM]; light blue) and *Small Cell* (B-cell lymphoma [DLBC], mesothelioma [MESO], sarcomas [SARC] and testicular germ cell tumors [TGCT]; blue).

To identify signatures associated with each cluster, we used ROC curves and calculated prediction power based on the area under the curve (Figures 1C, S2, and S3). This led to the identification of genes and pathways that were specific for each cluster: *MUC16* (CA125), specifically expressed in *Gynecological cancers*, and “TERMINAL_FUCOSYLATION,” specific for *Gastro-Intestinal cancers*, which includes enzymes involved in the synthesis of Lewis antigens, as sialyl-Lewis^a^ (sLe^a^, CA19-9). This suggests that some of the novel signatures identified in this analysis could also serve as potential biomarkers for the different cancer types, for example: Galectin-7 (encoded by the genes *LGALS7, LGALS7B*) for squamous cell carcinomas; *MUC21* for lung adenocarcinomas, *ST3GAL4* for melanomas, *MUCL1* for breast cancer (No TNBC), *FUT9* for brain cancers, and *FUT7* for leukemia (Figure 1C).

To explore the use of the GRGs and GRPs expression signatures as potential biomarkers, we shifted our focus to the group of cancers arising from the same tissue that ended up in different clusters based on the expression of GRGs (Figures S1 and S4). With the exception of Breast cancer (which is separated into TNBC and non-TNBC), the samples in these tissues are grouped based on their histological subtypes, separating SCC (all of which are clustered together) from other subtypes, such as adenocarcinomas. In esophagus carcinoma (ESCA), adenocarcinoma samples cluster with the *Gastro-Intestinal cancers*, while in lung and cervical cancer, adenocarcinomas are grouped together (Figure S4). In bladder cancer (BLCA), the *Luminal* molecular subtype forms its own cluster and the *Neural* is associated with the *Mixed – Small Cell* (Figure S4). These results support the idea that our analysis can serve as a resource for the potential discovery of new diagnostic biomarkers.

### Cancer cells are the main contributors to the tumor glycosylation signature

**Figure 2:**
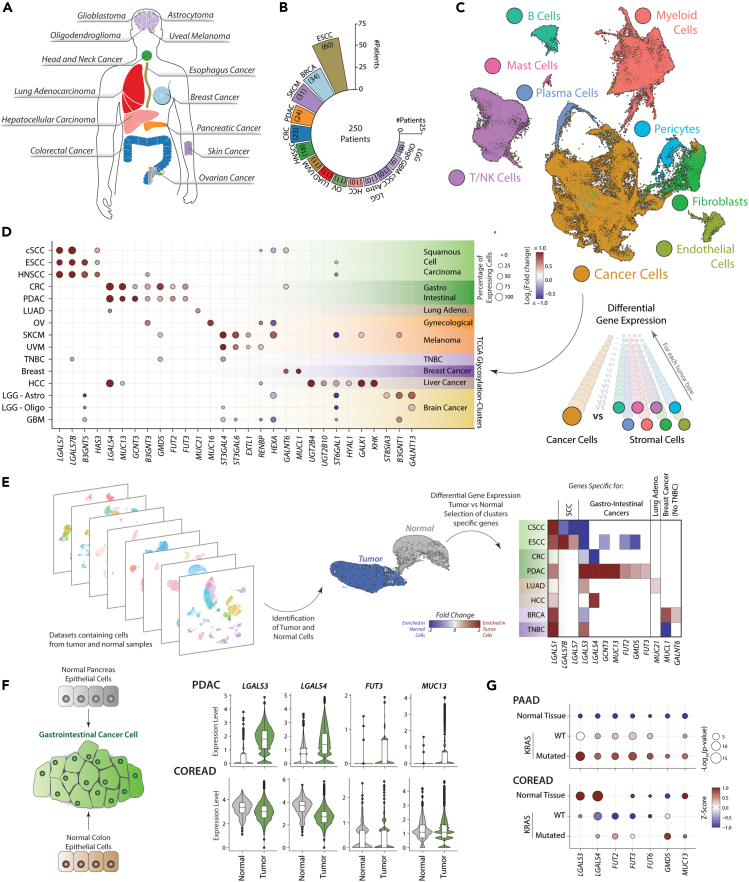
Cancer cells are main contributors to the specific tumor GRG expression signatures and is dependent on the cell of origin (A) Tumor types analyzed using single cell transcriptomics (scRNA-Seq). (B) Number of patients analyzed in the different tumor types. (C) UMAP plot showing the integrated scRNA-Seq dataset, colored based on cell types. (D) Dot Plot of selected differentially expressed GRGs between cancer and stromal cells in the integrated scRNA-Seq. Size correspond to the percentage of cells expressing each given gene and color is based on the fold change observed between Cancer and stromal cells. (E) Differential expression analysis of GRGs between normal and cancer cells in individual scRNA-Seq datasets. (F) Violin plot showing the expression of selected GRGs in Normal and Tumor cells from Pancreatic (PDAC) and Colorectal (COREAD) cancer. Data presented as boxplot indicate the median, 25th and 75th percentiles (hinges) and whiskers represent 1.5 times the interquantile range. (G) Dot plot displaying the differences between Normal and cancer tissue with or without mutations in KRAS. Statistical differences were evaluated using the Kruskal-Wallis test of one condition against the others.

The limitation of analyzing bulk transcriptomic data is the lack of cellular resolution for determining the relative contribution of the different cell populations to the tumor glycosylation patterns. To overcome this limitation, we analyzed single cell RNA-Seq (scRNA-Seq) from 14 different tumor types, representing 9 different TCGA glycosylation clusters and containing data from 250 patients (Figures 2A and 2B).^20^^,^^21^^,^^22^^,^^23^^,^^24^^,^^25^^,^^26^^,^^27^^,^^28^^,^^29^^,^^30^^,^^31^^,^^32^^,^^33^^,^^34^ These datasets were individually pre-processed, down sampled to 10.000 cells, and integrated into a single dataset using the Reciprocal principal component analysis (RCPA) algorithm as implemented in the package *Seurat*.^35^ Dimensionality reduction and clustering of this integrated dataset led to the identification of common populations, as immune cells (T and B lymphocytes, myeloid, and mast cells), other stromal cells (as fibroblasts, pericytes, and endothelial cells) and a cluster that contained malignant cells and some tissue-specific cells (Figures 2C and S5). To analyze the relative contribution of the cancer and stromal compartment to the overall expression of glycosylation-related genes in each tumor type, we performed differential gene expression between malignant cells and all the other cell types present in tissue (Figures 2D and S6). This analysis led to the identification of genes generally associated with cancer cells (*MUC1*, *LGALS3*, *UGDH*, *TSTA3,* and *GALE*) or the stromal compartment (*LGALS1*, *PSAP*, *LGALS9*, *IDS*, and *MGAT1*) in most of the tumor types analyzed (Figure S6). Interestingly, we observed that despite the strong association of Galectin-1 (*LGALS1*) and *PSAP* with the stromal compartment, cancer cells appear to be the main source expressing these genes in skin cutaneous melanoma (Figure S6). We also found GRGs that are associated with cancer cells in a tissue specific manner, including some of the genes that were identified in our previous analysis in bulk transcriptomics (Figures 2D and S6). For example, galectin-7 genes (*LGALS7* and *LGALS7B*) are expressed by cancer cells in SCC; fucosylation-related genes (*GMDS*, *FUT2*, *FUT3*) in Gastro-Intestinal cancers; *MUC21* in Lung Adenocarcinoma; *MUC16* in ovarian cancer; *ST3GAL4* and *ST3GAL6* in cutaneous and uveal Melanoma; *GALNT6* and *MUCL1* for non-TNBC breast cancer and *ST6GAL1* and *UGT2B4* for Liver Cancer (Figure 2D). These data show that cancer cells are the main contributors to the specific tumor glycosylation signatures found in tissue.

The strong association of TCGA glycosylation clusters with tumor type and the main contribution of cancer cells to their specific expression patterns, raises the question whether the cell of origin is a major determinant of cancer glycosylation signatures. To study this, we analyzed the expression of specific glycosylation-related genes in normal and cancer cells from tumors whose cell of origin has been identified. In cutaneous SCC, that originates from keratinocytes in the skin, we observed the expression of galectin-7 genes (*LGALS7* and *LGALS7B*) in both normal and cancer cells (Figure S7A). The same is the case for epithelial cells from normal colon or colorectal cancer, which express Galectin-3 (*LGALS3*) and 4 (*LGALS4*); hepatocytes from normal tissue and hepatocellular carcinoma (HCC) express *ST6GAL1* and *UGT2B4*; and melanocytes derived from cutaneous SCC (healthy) and cutaneous Melanoma (malignant), that both show the expression of *ST3GAL4* and *ST3GAL6* (Figure S7A). These data suggest that the cell of origin influences the GRG expression pattern of cancer cells and highlights the importance of studying changes in the expression of glycosylation genes between normal and cancer cells.

We next performed a differential gene expression analysis between cancer and normal cells in single cell RNA-Seq data containing samples from healthy and tumor tissue (Figures 2E and S6B).^21^^,^^26^^,^^28^^,^^31^^,^^32^^,^^33^^,^^34^ This allowed us to identify changes in GRG expression patterns specific for cancer cells, without being influenced by the different cell distributions that can be present in bulk transcriptomics. The gene *LGALS1* was found as the most commonly upregulated transcript in cancer cells of different tumor types (Figure 2E). We also found that some GRGs specifically associated with different tumor types are upregulated when compared to normal cells, as is the case of *MUC21* in lung adenocarcinoma and *GALNT6* and *MUCL1* in non-TNBC breast cancer. However, for some GRGs, the changes observed in the expression of GRGs depend on the tissue of origin. That is the case for genes encoding for galectin-7 (*LGALS7* and *LGALS7B*), highly expressed in SCC, that were found to be downregulated in cancer cells from cutaneous SCC but upregulated in the ones from esophagus SCC (Figure 2E). The same is the case for Galectin-3 (*LGALS3*) and Galectin-4 (*LGALS4*), associated with gastro-intestinal cancers, which are downregulated in colorectal (COREAD) but upregulated in pancreatic cancer (PAAD). Other GRGs highly expressed in gastro-intestinal cancers (as *GCNT3*, *GMDS*, *FUT2*, *FUT3*, and *MUC13*) are only found differentially upregulated in PAAD but not in COREAD (Figure 2E). This shows that despite that gastro-intestinal cancers present similar patterns of expression of GRGs, their expression dynamics from normal cells depend on the cell of origin (Figure 2F). We hypothesized that the presence of shared oncogenic pathways in these tumor types drive malignant cells to express a similar profile of GRG independent of the level observed in the cell of origin. Given that PAAD and COREAD are characterized by the presence of KRAS mutations, we next used the TCGA dataset to analyze if their presence in tissue is associated with changes in the expression of the GRGs specific for gastro-intestinal cancers.^36^ Similar to our analysis of scRNA-seq data, we observed that these GRGs are highly expressed in normal colon-rectum tissue and generally are downregulated or remain unchanged with malignant transformation (Figures 2G and S8). However, in pancreatic cancer these genes were found to be consistently upregulated when compared to normal tissue, an effect that was stronger in samples with KRAS mutations (Figures 2G and S8). Altogether, our data suggest that oncogenic pathways can lead to distinct changes in glycosylation profiles depending on the cell of origin.

### The contribution of the stromal compartment to the glycosylation-related gene expression profile

**Figure 3:**
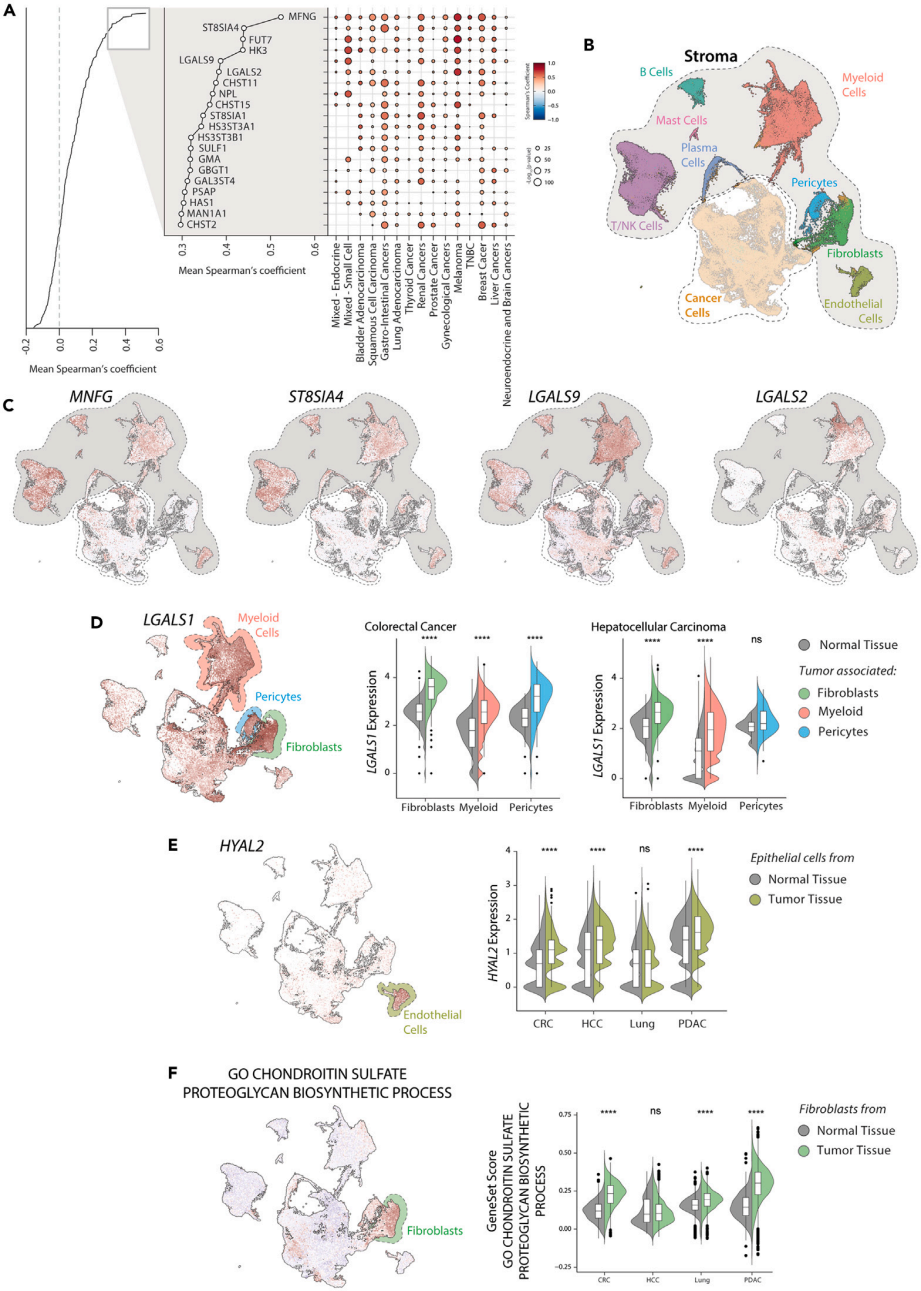
Stromal contribution to the tumor glycosylation (A) Correlation of GRGs with the stromal fraction in the TCGA dataset. (B) UMAP plot of the integrated scRNA-Seq dataset displaying the stromal cells. (C) Expression of the stromal associated genes *MNFG*, *ST8SIA4*, *LGALS9* and *LGALS2* in the scRNA-Seq integrated dataset. (D) UMAP plot showing the expression of *LGALS1* and Violin Plot illustrating its expression of *LGALS1* in Fibroblasts, Myeloid and Pericytes from normal or tumor tissue of Colorectal cancer and Hepatocellular carcinoma. (E) UMAP plot showing the expression of *HYAL2* and Violin Plot illustrating its expression of *HYAL2* in Endothelial cells from normal or tumor tissue in different tumor types. (F) UMAP plot showing the expression of the gene set “*Chondroitin sulfate - Proteoglycan biosynthetic process*” and Violin Plot illustrating its expression in Fibroblasts from normal or tumor tissue in different tumor types. Statistics: Wilcoxon test (∗p ≤ 0.05; ∗∗p ≤ 0.01; ∗∗∗p ≤ 0.001; ∗∗∗∗p ≤ 0.0001). In violin plot: data presented as boxplot indicate the median, 25th and 75th percentiles (hinges) and whiskers represent 1.5 times the interquantile range.

**Figure 4:**
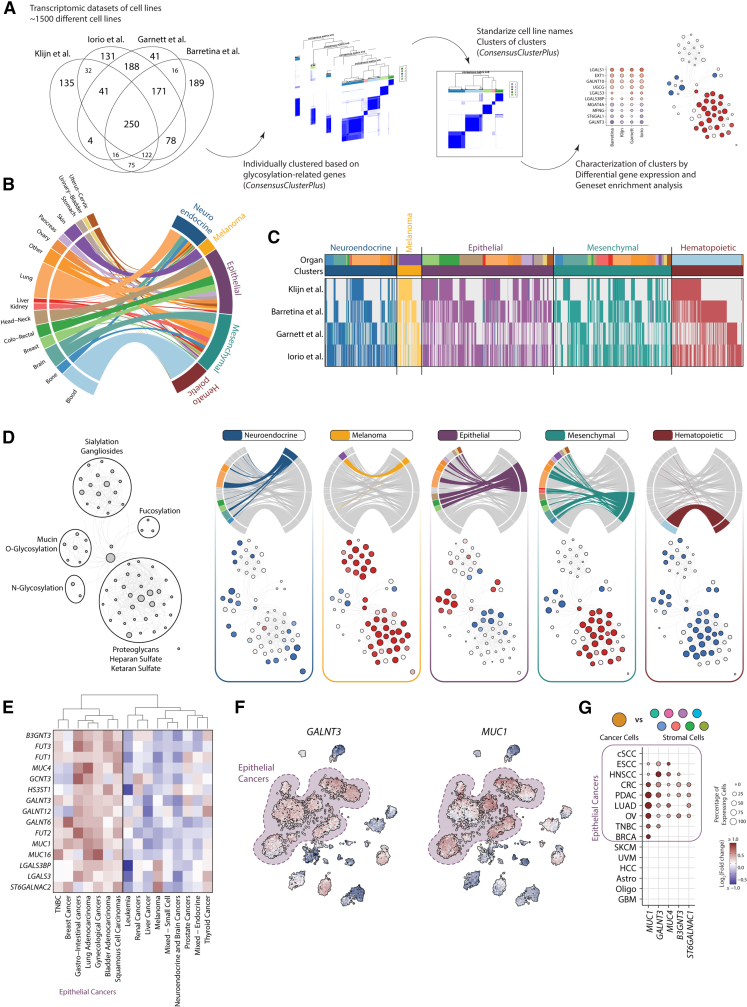
Cancer cell lines partially reflect the GRG expression signatures found in tissue (A) Workflow for the analysis of glycosylation clusters in cancer cell lines. (B) Chord diagram showing the association of the glycosylation clusters with the organ of origin of the cell lines. (C) Heatmap of cell lines membership to each glycosylation cancer in the different datasets. (D) Network representation of the gene set enrichment analysis (GSEA) results using Enrichment Map. Gene sets enriched in a given clusters are represented in red, while the ones downregulated are shown in blue. (E) Heatmap of GRGs associated with *Epithelial* cell lines in the different tissue meta-clusters. (F) TSNE plot showing the expression of *GALNT3* and *MUC1* in tissue samples of the TCGA dataset. (G) Dot plot representing the differential expression of GRGs associated with epithelial cell lines in cancer cells vs. stromal cells scRNA-Seq. Only significative differentially expressed genes are displayed.

Despite the fact that cancer cells are the main source of the specific GRGs and GRPs found in different tumor types, our results also show that the stromal compartment can contribute to the glycosylation profile present in the tissue (Figure S6). To confirm if what is observed in the scRNA-Seq analysis is also reflected in the different tumor types present in the TCGA dataset, we performed correlation analysis the expression of GRGs with the stromal fraction as previously described (Figure 3A).^37^ We confirmed that the stroma-associated genes identified in the previous analysis (as *LGALS1*, *PSAP*, *LGALS9,* and *IDS*; Figure S6) were also positively correlated with the stroma fraction in bulk RNA-Seq (Figure S9A). However, in this new analysis, we were able to identify other stroma-associated GRGs, such as *FUT7*, *HK3*, *LGALS2,* and *CHST11* (Figure 3A). If we plot the expression of some genes in the integrated scRNA-Seq dataset, we can observe different patterns of expression, with *MNFG* and *ST8SIA4* found in immune cells and *LGALS9* and *LGALS2* mainly restricted to myeloid cells (Figures 4B and 4C). These results suggest that different stromal cells present in tumor tissue have particular expression profiles of GRGs.

We next characterized the GRGs expressed by the different stromal cells using a modified version of the function *FindConservedMarkers* of the package *Seurat* to perform differential gene expression analysis in the integrated scRNA-Seq dataset (Figures S9B and S9C). This allowed us to identify the specific expression of *CHPF*, *UGDH,* and *HEXB* in fibroblasts; *HYAL2*, *GALNT18,* and *SULF2* in endothelial cells; *PSAP*, *LGALS9*, *MGAT1,* and *NPL* in myeloid cells; *MGAT4A* in T/NK Cells; *GALC* in mast cells and *ST6GAL1* in B and Plasma cells (Figure S9C). We also observed GRGs that are shared by different cell types, such as *SUFL1*, *LGALS3BP, UCGC* and *ST6GALNAC6* in pericytes and fibroblasts or *IDS* in B and T lymphocytes (Figure S9C). *LGALS1* was found to be expressed by fibroblasts, pericytes, and myeloid cells, all of which upregulate its expression in cancer compared to normal tissue (Figure 3D). The hyaluronidase 2 (*HYAL2*) is specifically expressed in endothelial cells and upregulated in different types of cancer (Figure 3E). Given that most of the GRGs found in pericytes seem to be present also in fibroblasts, we focused on the genes specifically found in the latter to better characterize their contribution to the overall expression of GRGs in the tumor. Interestingly, most of the GRGs that characterize fibroblasts (as *HEXA/B, CHSY1, CSGALNACT1/2, CHPF,* and *DSEL*) are associated with proteoglycan metabolism, specifically of chondroitin and dermatan sulfate. To confirm the specific expression of this pathway in fibroblasts, we calculated a score for the gene set GO term “Chondroitin sulfate - Proteoglycan biosynthetic process” in the scRNA-seq integrated dataset. We found that this pathway is greatly associated with Fibroblasts, which they upregulate in the tumor compared to normal tissue (Figure 3F). Our data thus shows that stromal and immune cells can also contribute to the expression profile of GRGs and GRPs present in tissue, but in a rather conserved manner between the various malignancies analyzed.

### Cancer cell lines partially reflect the tumor glycosylation patterns found in tissue

Cancer cell lines are *in vitro* tools that have been widely used for basic research, drug discovery, and glycomic analysis. As cancer cells are the main contributors to the specific tumor glycosylation signatures, we next analyzed if these patterns could be recapitulated in cancer cell lines. For this, we used four transcriptomic datasets that collectively represent 1489 cell lines that were individually clustered using the GRGs (Figure 4A).^38^^,^^39^^,^^40^^,^^41^ Given that the same cell line could be clustered differently according to the dataset, we performed a meta-cluster as previously described, which allowed us to find consensus clusters while conserving the diversity of the individual datasets (Table S4; Figure S10A). This strategy led to the identification of five GRG expression clusters in cancer cell lines, which were further characterized by performing gene set enrichment analysis (GSEA) and differential gene expression (Figures 4A–4C, S10B, S10C, S11A, and S11B).

Two of the clusters are associated with specific organs and represent the cell lines derived from *Melanoma* and *Hematological* cancers. These clusters are enriched with the gene sets associated with pigmentation and immune responses, respectively (Figure S11A). Similar to our findings tissue, melanoma cell lines are enriched in the expression of genes associated with the metabolism of sialic acid, including the sialyltransferases *ST3GAL4* and *ST3GAL6* (Figure 4D). The *Neuroendocrine* cluster is associated with neurotransmission gene sets and is mainly composed of cell lines from small cell lung cancer, neuroblastoma, and Ewing sarcoma (Figure 4B; Table S4). No specific glycosylation pathway was associated with this cluster, although they express the gene *FUT9*, which was overexpressed in brain cancers in bulk transcriptomic analysis (Figures 4D and S2). We also found two heterogeneous clusters that contain cell lines derived from different organs, which we named *Epithelial* and *Mesenchymal* given that they are enriched in gene sets associated with cell-to-cell contact and epithelial to mesenchymal transition (EMT), respectively (Figure S9D). Our results show that *epithelial* cell lines are particularly enriched in the expression of transcripts and gene transcript sets associated with mucin-type *O*-glycosylation and fucosylation (Figures 4D and S11B). The most overexpressed gene transcript in this cluster was *GALNT3*, which has been linked to maintaining the epithelial phenotype in different contexts.^13^^,^^42^ On the other hand, *mesenchymal* cells are correlated with the expression of genes involved in proteoglycan metabolism and overexpress the gene encoding for Galectin-1 (*LGALS1*, Figure S11B).

These data suggest that cancer cell lines present a simplified landscape of GRGs compared to the ones found in tissue. However, given that our results can be influenced by the tumor types present in each dataset and the difference in the number of cell lines and tissue analyzed, we investigated if similar patterns of GRG expression can also be found in the TCGA dataset. For this, we generated metaclusters by performing hierarchical clustering using the median expression of each GRG in the clusters identified in Figure 1. Two main metaclusters were found, one of which contains most of the cancers classically associated with an epithelial phenotype (gastro-intestinal, lung, gynecological, breast and squamous cell cancers; Figure S11C). These results are in line with our previous analysis, which showed that the cell lines derived from pancreas, colorectal, stomach, head and neck, ovary, and breast cancers are grouped together with the epithelial cluster, while the ones from kidney, liver, and brain cancer presented a mesenchymal phenotype (Figure S10C). The epithelial tissue metacluster showed an expression profile similar to the epithelial cluster found in cell lines, including *GALNT3* and *MUC1* (Figures 4E and 4F). Similar results were found in the analysis of single cell transcriptomics (Figure 4G). We next checked if cell lines can still show the expression of tissue-specific GRGs, despite not forming an independent cluster (Figures S11D‒S11I). Similarly, to the profiles identified in tissue, we observed the expression of terminal fucosylation genes in cell lines from Gastro-intestinal cancer, *HAS3* and *LGALS7* in SCC and *MUCL1* in non-TNBC Breast cancer (Figures S11D‒S11G). However, we did not observe the association of *ST6GAL1* with Liver cancer or *ST8SIA1* with brain cancers (Figures S11H‒S11I).

As EMT can be considered as a spectrum rather than discrete phenotypes, we next study how the expression of different GRG and GRP is associated with a previously defined EMT score in cell lines from epithelial cancers (Figures S12A‒S12C).^43^^,^^44^ As anticipated, we observed a negative correlation with GRG associated with an epithelial phenotype (*ST6GALNAC1*, *FUT3*, *GALNT3*, B3GNT3), while a positive correlation with mesenchymal genes (*LGALS1*, *EXT1*, *UGCG*) (Figure S12A). When analyzing GRPs, we found that EMT was associated with an increase in the expression of the biosynthetic pathways for chondroitin sulfate and gangliosides and a decrease in genes associated with *O*-glycans and fucosylation (Figures S12B‒S12C). The upregulation of gangliosides and downregulation *O*-glycosylation and terminal fucosylation during EMT had been previously reported.^13^^,^^45^ To study if these results are also reflected in patients, we performed a similar analysis in the scRNA-Seq data, using the epithelial cells identified in tumor samples from pancreatic, colorectal, and lung cancer. Our findings reveal a negative correlation between *O*-glycosylation and terminal fucosylation with the EMT score, in similar fashion as in cell lines, although this effect was not observed for lung cancer (Figure S12D). On the other hand, there was not a clear association between the EMT score with the GRP for the biosynthesis of chondroitin sulfate and gangliosides, except for a modest positive correlation in pancreatic cancer. In summary, our results underscore that cancer cell lines only partially replicate the GRG profile observed in tissue, emphasizing the nuanced relationship between glycan-related processes and the dynamic spectrum of EMT in cancer.

### Association of glycosylation-related gene expression patterns with the clinical outcome of patients

**Figure 5:**
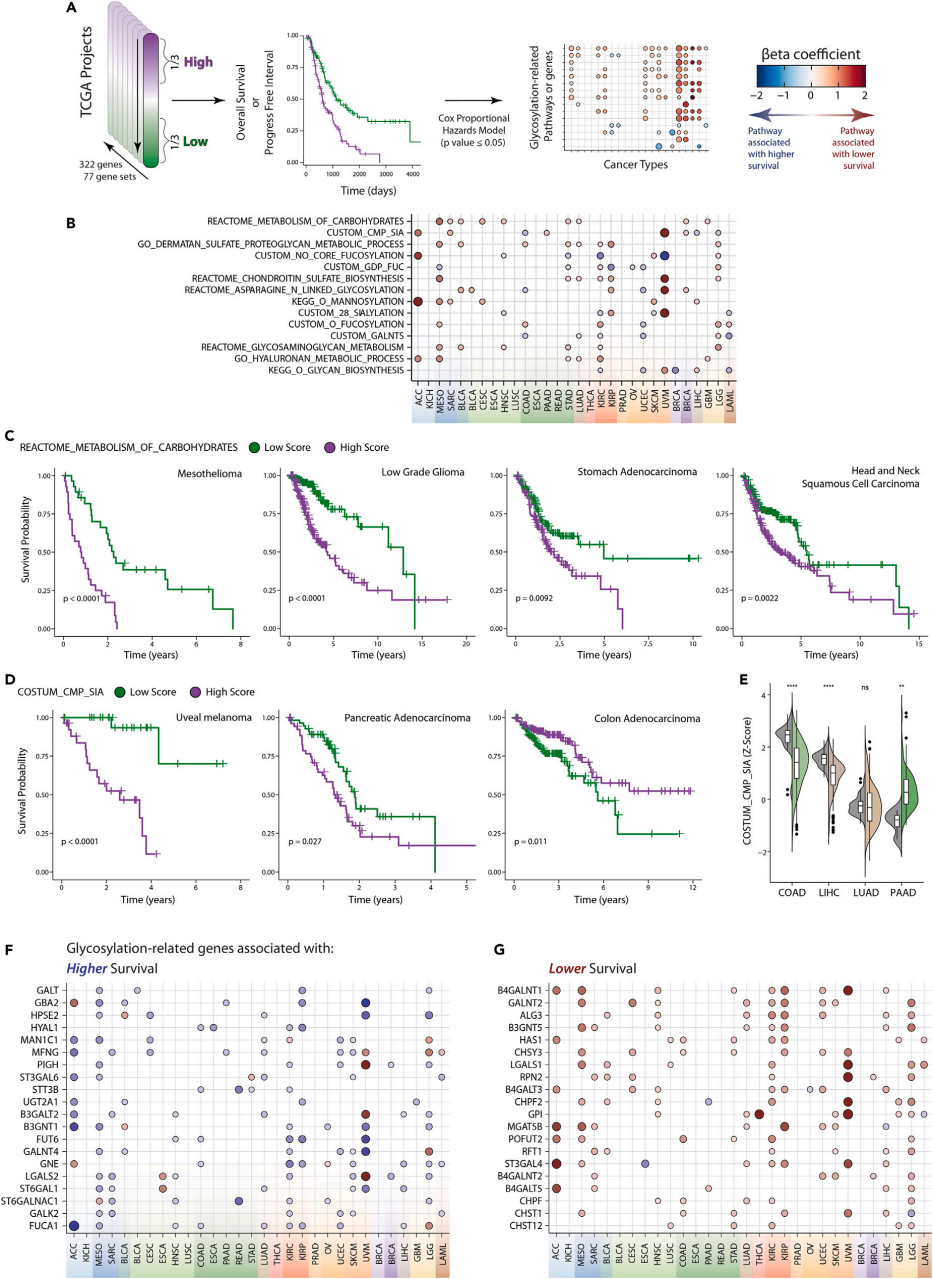
Association of different glycosylation pathways with clinical outcome of patients (A) Workflow used to study the association of glycosylation-related genes and pathways with survival. (B) Dot plot summarizing the association of glycosylation-pathways with survival. Kaplan-meier curves illustrating the survival of patients that present a high or low score for the Reactome gene set “Metabolism of Carbohydrates” (C) and the custom gene set “Sialic acid – CMP synthesis” (D) in different tumor types. Statistics: Log rank test. (E) Expression of the custom dataset “Sialic acid – CMP synthesis” in normal (gray) and tumor (color) tissue. Statistics: Wilcoxon test (∗p ≤ 0.05; ∗∗p ≤ 0.01; ∗∗∗p ≤ 0.001; ∗∗∗∗p ≤ 0.0001). In violin plot: data presented as boxplot indicate the median, 25th and 75th percentiles (hinges) and whiskers represent 1.5 times the interquantile range. Dot plot of summarizing the results of the survival analysis of glycosylation-related genes mostly associated with higher (F) or lower (G) survival.

We next investigated if the expression of different glycosylation-related genes and pathways was associated with clinical outcome. To analyze this, we performed a systematic survival analysis in the TCGA dataset by grouping the patients based on the GRGs and GRPs expression in the top or bottom third and studying the difference in survival rate between these groups using Kaplan-Meier curves and statistical analysis using the Cox proportional hazard model (Figure 5A). This analysis was repeated for each combination of glycosylation cluster and TCGA project (Figures 5A, 5B, and S13).

The glycosylation pathway associated with more aggressive cancer was the Reactome gene set “Metabolism of carbohydrates,” in agreement with previously published reports (Figures 5B and 5C).^46^ This gene set includes a great variety of genes encoding for glycoproteins, transporters, and enzymes related to glycosylation and general carbohydrate metabolism, suggesting that a general upregulation of the pathways associated with glycan metabolism is associated with tumor progression (Figures 5B and 5C). Pathways corresponding to the biosynthesis of different glycosaminoglycans (dermatan sulfate, chondroitin sulfate, and hyaluronan) were also associated with a lower survival rate in patients with cancer (Figure 5B). This may reflect the role of fibroblast activation in cancer progression, given that glycosaminoglycans-related genes are mainly expressed in this cell type and upregulated in tumor tissue (Figure 3F).

The association of some glycosylation pathways with survival depends on the cancer type, as is the case for “*Synthesis of CMP-Sialic acid*” or “*Terminal fucosylation*” (Figure 5B). For example, the expression of the gene set reflecting the “*Synthesis of CMP-Sialic acid*” is associated with better survival in colorectal and liver cancer, but with lower survival in sarcoma, uveal melanoma and lung, pancreatic and breast cancer (Figures 5B–5D). Remarkably, this seems to be related to the expression in normal tissue (Figure 5E). This pathway is associated with better survival in cancers of organs where is highly expressed in normal tissue and presents a downregulation in cancer samples (as in the colon and liver), while the opposite is observed in pancreatic cancer (Figure 5E). We repeated this analysis to study the correlation of genes with survival, which led to the identification of a series of genes whose expression is associated with lower or higher survival in different cancer types (Figures 5F and 5G). Intriguingly, some of the genes associated with a better survival are associated with different immune populations, such as *MFGN* and *LGALS2* (Figure 3C), suggesting that these results reflect the anti-tumor immune responses (Figure 5F). On the other hand, some glycosylation-related genes associated with lower survival can be related to the induction of regulatory immune responses, as is the case of LGALS1 (known by its immunomodulatory properties) and ST3GAL4 (one of the enzymes responsible for the synthesis of Siglec-9 ligands).^9^^,^^47^

In conclusion, this analysis allowed us to identify associations between glycosylation-related genes and pathways and survival, showing that some associations seem to be conserved between different tumor types, while others are highly dependent on the tumor type.

## Discussion

In this article, we performed a deep characterization of the transcriptional landscape of GRGs and GRPs in a pan-cancer setting. The clustering of samples based on GRGs revealed patterns of expression that were highly dominated by the cell ontogeny and the tumor type, similar to previous pan-cancer studies.^10^ However, we also observed shared patterns of GRGs among different tumor types, as is the case for gastro-intestinal cancers and squamous cell carcinomas, which cluster together independent of the organ of origin. Moreover, our results show that the TNBC cluster separated from other types of breast cancer, illustrating their unique GRG expression patterns.

The identification of different GRGs and GRPs enriched in different glycosylation clusters can serve as a source for the discovery of new diagnostic biomarkers. This is supported by the fact that some of the genes and pathways identified in our analysis are associated with biomarkers currently used in the clinic, as is the case with MUC16 for gynecological cancers or CA19-9 (sialyl Lewis^A^, a fucosylated antigen) for gastro-intestinal cancers.^2^^,^^6^^,^^7^ Moreover, we also identified other clinically relevant GRGs, as is the case of *ST8SIA1* and *B4GALNT1*, which contribute to the synthesis of ganglioside GD2, in brain cancers. GD2 is abundantly expressed in some brain tumors and has been considered an interesting target for directed anti-tumor therapies.^48^^,^^49^ Therefore, the data presented here can also serve as a resource for the identification of novel diagnostic biomarkers or potential therapeutic targets, as galectin-7 (*LGALS7*) for Squamous cell carcinomas, galectin-4 (*LGALS4*) for gastro-intestinal cancers, *MUC21* for lung adenocarcinoma, *MUC15* for thyroid cancer and *MUCL1* for breast cancer.

Given that several tumor-associated glycans have been correlated with the induction of a tolerogenic microenvironment, the identification of specific GRGs and GRPs can aid in better understanding glycan-mediated regulatory programs in cancer.^3^^,^^50^ Gastro-intestinal cancers are highly enriched in the expression of fucosyltransferases responsible for the synthesis of Lewis antigens, suggesting that these tumor types may be particularly affected by the fucose-specific immunomodulatory receptor DC-SIGN (dendritic cell-specific intercellular adhesion molecule-3-grabbing non-integrin). Triggering of DC-SIGN on antigen-presenting cells by fucose-containing glycans induces the expression of IL-10 and enhances the capacity to differentiate Th2 cells.^13^^,^^51^ DC-SIGN ligands present in colorectal cancer cell lines suppress dendritic cell function and differentiation.^52^^,^^53^ In pancreatic cancer, the expression of fucosylation-related genes by cancer cells is correlated with a tolerogenic phenotype in macrophages.^13^ Moreover, the presence of DC-SIGN^+^ macrophages is associated with a suppressive microenvironment and poor clinical outcomes in gastric cancers.^54^ Brain cancers are characterized by a high expression of the α1-3 fucosyltransferase FUT9, which has been shown to catalyze the synthesis of the Lewis^X^ antigen in mouse models.^55^ We previously reported that human myelin is decorated with Lewis^X^ antigens that mediate the interaction with DC-SIGN^+^ microglia, inducing a immunoregulatory program that contributes to the immune homeostasis in healthy brain.^56^ However, its expression in brain cancer may contribute to the tolerogenic microenvironment.

Sialylated glycans are known for their immunomodulatory properties and have been described as a self-associated molecular pattern.^57^^,^^58^^,^^59^^,^^60^ Sialic acids are linked to the underlying glycan structures in different linkages (namely α2-3, α2-6, and α2-8), each of which is catalyzed by a group of different enzymes.^57^ Here we describe that enzymes that catalyze α2-3 sialylation are associated with Melanoma, α2-6 sialylation with thyroid cancer and α2-8 sialylation (also called polysialylation) with brain cancers. Sialylated glycans can be recognized by a family of lectin receptors called Siglecs (sialic acid-binding immunoglobulin-like lectins), most of which present an ITIM (immunoreceptor tyrosine-based inhibitory motif) sequence in their intracellular domain.^57^ It was previously reported that ligands of Siglec-7 and Siglec-9 can be found in cancer cells and cell lines from different tumor types, but are particularly increased in melanoma.^3^^,^^58^ This is in line with our results that melanoma tissue samples and cell lines highly express *ST3GAL4*, which is known to synthesize the ligands of Siglec-9.^47^^,^^59^

The use of scRNA-Seq datasets allowed us to resolve the expression of GRGs at a single cell level, identifying cancer cells as the main contributors to the specific genes observed in the analysis of bulk transcriptomic data. Our results suggest that the expression dynamics of GRGs and GRPs in tumor development is influenced by the cell of origin and the oncogenic pathways that lead up to malignant transformation. For example, pancreatic and colorectal cancer cells, despite having a similar expression profile of GRGs and GRPs, present different changes with respect to their normal counterparts. We hypothesize that KRAS mutations can push cells to a similar glyco-phenotype independent of the cell of origin, given that it is an oncogenic driver commonly found in both cancer types.^36^ It has been previously shown that KRAS mutations can lead to different signaling pathways and transcriptomic changes in a tissue-specific manner.^61^^,^^62^ The complexity of oncogenic pathways that are present in cancer hinders the systematic study in a pan-cancer setting and highlights the need to study their effect on the expression of GRGs and GRPs in the context of specific organs or cancer types, considering also clinically relevant variables.

Despite the cancer cells seem to be responsible for the expression of specific GRGs and GRPs in each tumor type, stromal cells can also contribute to the overall tumor glyco-code, although in a rather conserved form across different tumor types. For example, Galectin-1 (*LGALS1*) was mainly expressed by fibroblasts, pericytes, and myeloid cells, all of which upregulate its expression in tumor compared to normal tissue. Stromal-derived Galectin-1 has been associated with vascularization and metastasis formation in pancreatic cancer, proving to be a good candidate for therapeutic targeting.^63^ Tumor-associated endothelial cells appear to increase the expression of hyaluronidase 2 (*HYAL2*), one of the enzymes responsible for the degradation of hyaluronan, the main component of the endothelial glycocalyx.^64^ This process leads to the generation of the pro-tumoral mediator low-molecular-weight hyaluronan, which can induce inflammation, immune cell recruitment and promote the migration of cancer cells.^65^^,^^66^ The characterization of the stromal contribution to the overall profile of GRGs and GRPs found in tumor tissue is essential for the correct interpretation of the results obtained with the analysis of bulk transcriptomics.

Cell lines are an important tool in cancer research, although several studies have shown that they differ in their ability to reflect the primary tumor.^67^^,^^68^^,^^69^^,^^70^ The clustering of cell lines based on GRGs led to the identification of two general clusters, epithelial and mesenchymal, that correspond to most of the cell lines and three tissue-specific clusters (melanoma, neuroendocrine, and hematopoietic). Of these clusters, melanoma cell lines seem to represent better the GRGs and GRPs present in the tissue, characterized by a high expression of sialylation-related genes. Klijn et al. have shown that the unsupervised clustering of cell lines is dominated by the epithelial-to-mesenchymal transition (EMT), which is in line with our results.^40^ Epithelial cancer cell lines and tissues are characterized by the expression of genes associated with Mucin-type O-glycosylation and terminal fucosylation, similarly to our prior results in pancreatic cancer.^13^ One of the genes associated with these cell lines is *GALNT3*, which has been associated with the epithelial phenotype in different contexts.^13^^,^^42^ Conversely, the fucosylation of TGFβ receptors in colorectal cancer cell lines by *FUT3* and *FUT6* is essential for the TGFβ-mediated induction of EMT, which suggests that the enrichment of fucose-related genes in epithelial cells “prepare” these cells to undergo to this transition.^71^ In mesenchymal cells, we found an enrichment of Galectin-1 (*LGALS1*), which has been associated with EMT in hepatocellular carcinoma, and an increase in genes associated with the biosynthetic pathway of gangliosides, which has been previously reported for ovarian cancer.^45^^,^^72^ However, EMT is now understood to be more nuanced than a binary switch between extreme phenotypes, with the existence of intermediate states.^43^^,^^73^ Moreover, the EMT programs that led to this differentiation are tissue- and context-dependent.^73^ Therefore, there is a need for a rigorous selection of the cell lines to be used in the study of GRGs and GRPs in cancer biology, based on their capacity to reflect the expression of the gene of interest in tissue. Moreover, it is important to note that glycosylation patterns can be influenced by culture conditions, as hypoxia or the presence of nutrients, and adding an extra layer of complexity that need to be considered.^74^^,^^75^

Finally, we identified GRGs and GRPs associated with the clinical outcome of patients, which indicate that changes in the tumor glyco-code affect disease progression. The transcriptomic analysis of GRGs led to the identification of tumor subtypes with differences in the overall survival in pancreatic and breast cancer.^12^^,^^13^ Interestingly, our analysis showed that the association of several pathways with survival depends on the tumor type, as is the case for “*Synthesis of CMP-Sialic acid*,” which leads to the generation of the glycan donor used by sialyltransferases. This pathway is associated with a worst survival uveal melanoma, pancreatic and lung cancer, while the opposite is observed in colorectal and liver cancer. We previously reported that the knockout of CMAS, a key enzyme in this pathway, leads to a more aggressive tumor in a mouse model of colorectal cancer.^76^

The characterization of the differential contribution of the tumor and stromal compartment to the overall expression profile allows for a more accurate interpretation of the results obtained from the analysis of bulk transcriptomics. Several of the GRGs and GRPs commonly correlated with survival in different tumor types are actually associated with specific cells in the stromal compartment (as *MFNG* in all immune cells, *LGALS2* in myeloid cells, *ST6GAL1* in B and plasma cells and *CHPF* and *ST3GAL4* for fibroblasts). This may reflect the presence of different cell populations when analyzing survival in bulk transcriptomics and does not necessarily reflect the role of the specific gene or pathway. The use of single cell and spatial techniques for the characterization of tissue samples can help to circumvent this issue and to full characterize the tumor glyco-code.

The transcriptional analysis of GRGs and GRPs is a powerful tool for a better understanding of glycosylation and different approaches have been undertaken to obtain novel insights into its role in health and disease.^4^^,^^13^^,^^77^ However, their potential to predict glycan structures has several limitations associated with the organization, complexity, and regulation of the glycosylation machinery. Alterations in tumor glycosylation can be the result of multiple mechanisms other than the expression of GRGs, including changes in the localization of glycosyltransferases or differences in chaperone function.^78^ The Tn antigen, one of the most studied glycan structures in cancer, corresponds to the first step of mucin-type *O*-glycosylation: a simple *N*-Acetyl-galactosamine bound to a serine, threonine, or tyrosine in the peptide backbone of a protein.^3^^,^^50^^,^^79^^,^^80^ Its synthesis is catalyzed by a family of twenty enzymes called polypeptide *N*-Acetylgalactosaminyltransferase (encoded by the genes *GALNT*) that differ in their tissue distribution and specificity.^81^^,^^82^ However, their presence in tumor cells is difficult to predict based only on transcriptomic analysis as it can be influenced by multiple factors: activity or defects of the extending enzymes (C1GALT1 and ST6GALNAC1) or the COSMC chaperone (C1GALT1C1), overexpression of carriers (such as mucins), and GALNT re-localization to the endoplasmic reticulum.^83^^,^^84^^,^^85^ Moreover, the organization of the glycosylation machinery as an “assembly line” complicates the prediction of glycan structures based on the transcriptomics analysis, as there is a need to not only know the expression of the gene of interest, but also all the others that contribute to the synthesis of their potential substrates.^86^ This is illustrated in colorectal cancer, where fucosylated glycans have been reported to be present in both normal and tumor tissue, in line with our results that show no changes in the expression of fucosyltransferases.^87^ However, differences in the specific fucose-containing structures have been recently reported, with cancer cells characterized by the synthesis of sialyl-Lewis core 2 *O*-glycans.^87^ This illustrates the need for a multidisciplinary approach to complete our understanding of the role of glycosylation in cancer, integrating transcriptomic and glycomic analysis with functional and clinical studies. Moreover, future efforts to correlate the presence of glycosylation structures within the whole transcriptome will shed a light on novel regulatory pathways and the potential use of gene expression for the prediction of the tumor glyco-code.

### Limitations of the study

The present article relies in the analysis of transcriptomic data to study the expression of glycosylation-related genes and pathways in cancer. Nevertheless, it is crucial to note that gene expression does not always correlate with the actual presence of proteins. This discrepancy is further deepened by the intricate nature of the glycosylation machinery, making it difficult to predict the presence of specific glycan structures. Moreover, the quality of the transcriptomic datasets used can also affect the results. Given the relatively low sensitivity of scRNA-Seq, low expressing GRGs are probably not detected, limiting our capacity to study them. The transcriptomic data from cancer cell lines analyzed in our article is also influenced by the culture conditions used by the different research groups.

## STAR★Methods

### Key resources table

**Table:** 

REAGENT or RESOURCE	SOURCE	IDENTIFIER
**Deposited data**		

TCGA pan cancer dataset	TCGA	https://gdc.cancer.gov/about-data/publications/pancanatlas
scRNA-Seq Data - Esophageal Squamous cell carcinoma	Zhang, X. et al.^33^	GSE160269
scRNA-Seq Data - Cutaneous Squamous cell carcinoma	Ji, A. L. et al.^27^	GSE144236
scRNA-Seq Data - Colorectal Cancer	Lee, H. O. et al.^28^	GSE132465
scRNA-Seq Data - Breast Cancer	Pal, B. et al.^34^	GSE161529
scRNA-Seq Data - Skin Melanoma	Sade-Feldman, M. et al.^23^	GSE120575
scRNA-Seq Data - Skin Melanoma	Jerby-Arnon, L. et al.^24^	GSE115978
scRNA-Seq Data - Uveal Melanoma	Durante, M. A. et al.^30^	GSE139829
scRNA-Seq Data - Ovarian Cancer	Izar, B. et al.^29^	GSE146026
scRNA-Seq Data - Glioblastoma	Neftel, C. et al.^25^	GSE131928
scRNA-Seq Data - Lung Cancer	Kim, N. et al.^31^	GSE131907
scRNA-Seq Data - Hepatocellular Carcinoma	Lu, Y. et al.^32^	GSE149614
scRNA-Seq Data - Head and neck Squamous cell carcinoma	Puram, S. V. et al.^21^	GSE103322
scRNA-Seq Data - Low grade glioma – Oligodendroglioma	Tirosh, I. et al.^29^	GSE70630
scRNA-Seq Data - Low grade glioma – Astrocytoma	Venteicher, A. S. et al.^22^	GSE89567
scRNA-Seq Data - Pancreatic ductal adenocarcinoma	Peng et al.^26^	PRJCA001063
Transcriptomic Cell lines	Barretina, J. et al.^38^	GSE36133
Transcriptomic Cell lines	Garnett, M. J. et al.^39^	E-MTAB-783
Transcriptomic Cell lines	Klijn, C. et al.^40^	E-MTAB-2706
Transcriptomic Cell lines	Iorio, F. et al.^41^	E-MTAB-3610

**Software and algorithms**		

*ConsensusClusterPlus*	Wilkerson, M.D. et al.^88^	https://bioconductor.org/packages/release/bioc/html/ConsensusClusterPlus.html
*GSVA*	Subramanian, A. et al.^18^	https://bioconductor.org/packages/release/bioc/html/GSVA.html
*survival*	Therneau, T. et al.	https://cran.r-project.org/web/packages/survival/index.html
*survminer*	Kassambara, A. et al.	https://cran.r-project.org/web/packages/survminer/index.html
Seurat (v4)	Hao, Y. et al.^35^	https://cran.r-project.org/web/packages/Seurat/index.html
Gene Set Enrichment Analysis - javaGSEA Desktop Application	Broad Institute	https://www.gsea-msigdb.org/gsea/index.jsp
Cytoscape	Shannon, P. et al.	cytoscape.org
EnrichmentMap	Merico, D. et al.^89^	https://enrichmentmap.readthedocs.io/en/docs-2.2/index.html
Limma	Ritchie, M. E. et al.^90^	https://bioconductor.org/packages/release/bioc/html/limma.html
Scripts used for the transcriptomic analysis	Present manuscript	https://github.com/MolecularCellBiologyImmunology/Glyco_PanCancer

**Other**		

Cellosaurus	Bairoch, A. et al.^91^	https://web.expasy.org/cellosaurus/

### Resource availability

#### Lead contact

Further information and requests for resources and reagents should be directed to and will be fulfilled by the lead contact, Prof. Dr. Yvette van Kooyk (y.vankooyk@amsterdamumc.nl).

#### Materials availability

This study did not generate new unique reagents.

#### Data and code availability

• TCGA pan cancer dataset was downloaded from https://gdc.cancer.gov/about-data/publications/pancanatlas. Single cell RNA-Seq datasets were downloaded from the NCBI GEO database (https://www.ncbi.nlm.nih.gov/geo/) with the following accession numbers: GSE160269 (Esophageal Squamous cell carcinoma, ESCC),^33^ GSE144236 (cutaneous Squamous cell carcinoma, cSCC),^27^ GSE132465 (Colorectal Cancer, COREAD),^28^ GSE161529 (Breast Cancer, BRCA),^34^ GSE120575 (Melanoma, SKCM),^23^ GSE115978 (Melanoma, SKCM),^24^ GSE139829 (Uveal melanoma, UVM),^30^ GSE146026 (Ovarian cancer, OV),^29^ GSE131928 (Glioblastoma, GBM),^25^ GSE131907 (Lung adenocarcinoma, LUAD),^31^ GSE149614 (Hepatocellular carcinoma, HCC),^32^ GSE103322 (Head and neck Squamous cell carcinoma, HNSCC),^21^ GSE70630 (Low grade glioma – Oligodendroglioma, LGG)^20^ and GSE89567 (Low grade glioma – Astrocytoma, LGG).^22^ For Pancreatic ductal adenocarcinoma (PAAD), Peng et al. was downloaded from the Genome Sequence Archive (https://bigd.big.ac.cn/gsa/) under the project PRJCA001063.^26^ The TCGA survival data was obtained from the publication of Liu et al. ^92^ and the mutational data from Sanchez-Vega et al.^36^ The datasets with transcriptomic data of cell lines were downloaded from the NCBI GEO database, accession number GSE36133,^38^ or from ArrayExpress (https://www.ebi.ac.uk/arrayexpress/), identifiers: E-MTAB-783,^39^ E-MTAB-2706^40^ and E-MTAB-3610.^41^
• All original code has been deposited at Github (https://github.com/MolecularCellBiologyImmunology/Glyco_PanCancer) and is publicly available as of the date of publication.
• Any additional information required to reanalyze the data reported in this paper is available from the lead contact upon request.

### Method details

#### Analysis TCGA dataset

In the pan-cancer TCGA dataset, we filtered out the low expressed genes by discarding the ones that present less than 5 counts in half of the samples. To analyze the presence of glycosylation profiles shared between the different cancer types, we used package *ConsensusClusterPlus*^88^ to perform a clustering using the median-centered expression of the 100 most variable glycosylation genes (defined by interquantile range, IQR) and the following settings: 500 iterations with 90% of sampling, “*hclust*” as method and “*ward*.*D2*” as inner and final linkage. For the gene set enrichment analysis, we used the method *ssGSEA* of the *GSVA* package.^93^ We used Receiver Operating Characteristic (ROC) curves to define genes and pathways associated with each cluster, calculating the prediction power from the area under the curve (AUC) as absolute(AUC-0.5)∗2. Genes and pathways with a prediction power equal or higher of 0.85 were selected as specific for each cluster.

For the systematic analysis of the association of gene expression with overall survival of patients, we selected the combination of TCGA Project with glycosylation clusters that present at least 60 patients. To analyze the association of different genes and pathways with overall survival, samples were divided in thirds based on gene expression and differences in the survival between top and bottom third was analyzed using the function *coxph* from the package *survival*, using the Cox proportional hazards regression model. Kaplan–Meier curves were generated using the function *ggsurvplot* (from the R package *survminer*). The assumption of the Cox model that hazards are proportional was validated using the function *cox.zph* from the R package *survival*, and conditions that failed this test were discarded from the final analysis.

#### scRNA-seq analysis

*Seurat(v4)* package was used for the analysis of single cell RNA-Seq.^35^ For each dataset, we generated Seurat objects with samples obtained from tumor tissue, downsampled to 10.000 cells and proceeded to their integration using Fast integration using reciprocal PCA (RPCA) method, following the recommendations of the developers. Clustering was performed using the functions *FindNeighbors* using the first 30 Principal Component Analysis (PCA) dimensions and *FindClusters* with resolution set as 1. Clusters were visualized using uniform manifold approximation and projection (UMAP), using the function *RunUMAP*. Cell identification was done based on gene expression and the cell types present in the original publications. In our analysis, we identified a cluster composed mainly of malignant cells and tissue-specific cell types, which we cleaned up to only select cancer cells. For each tumor type, we selected cells corresponding to this cluster and proceed to normalize and perform PCA. Presence of different cell types was analyzed by running the functions *FindNeighbors*, *FindClusters* and *RunUMAP* using the first 50 PCA components. Cells were discarded based on the expression of different lineage markers, as *PTPRC* for immune cells, *CD3G* and *CD8A* for T Cells, *MS4A1* and *CD19* for B Cells, *AIF1* and *CD14* for Myeloid cells, *LUM* for Fibroblasts, *RGS5* for Pericytes, *CDH5* for Endothelial cells, *PMEL* for Melanocytes, *MOG* and *MBP* for Oligodendrocytes or *GFAP* for Astrocytes. Differential gene expression in the integrated dataset was performed using custom-made function *FindMarkersPerTumorType*, modified from the function *FindConservedMarkers* of the *Seruat* package.

To analyze the differential gene expression between normal and cancer cells, we performed the scRNA-Seq analysis of the datasets from ESCC,^33^ cSCC,^27^ COREAD,^28^ BRCA,^34^ LUAD,^31^ HCC^32^ and PDAC.^26^ Data was normalized and scaled using the *SCTransform* function (regressing out the mitochondrial percentage), and Principal Component Analysis (PCA) was performed with the 3000 most variable genes. PCA components 1 to 30 were used for graphical based clustering at a resolution of 1. These clusters were projected onto Uniform Manifold Approximation and Projection (UMAP) dimensional reduction. Differential gene expression between normal and cancer cells was done using the function *FindMarkers*. For this analysis, we used epithelial cells in normal and tumor tissue for ESCC, COREAD, PDAC, LUAD and BRCA (identified by the expression of epithelial and cytokeratin genes, as *KRT19*, *EPCAM* and *CDH1*); Keratinocytes for cSCC (*COL17A1*, *KRT14* and *KRT10*) and Hepatocytes for HCC (*ALB*, *FGA* and *FGG*).

To study the correlation between glycosylation-related pathways with Epithelial to Mesenchymal transition (EMT), we selected epithelial cells, renormalize using the function *SCTranform* (regressing out percentage of mitochondrial genes) and calculate gene scores with the function *AddModuleScore*. An EMT score was calculated by performing the difference between an Epithelial and Mesenchymal Score, calculated using gene signatures previously described.^44^

#### Cell line transcriptomic analysis

Given that the datasets used in this paper sometimes provide slightly different names for the same cell lines, we first uniformized the designation by changing the characters to upper case and eliminating punctuation marks. Moreover, we checked for synonyms of different cell lines using the database Cellosaurus (https://web.expasy.org/cellosaurus/).^91^ This method led to 1489 unique cell lines (Table S4).

Each dataset was independently clustered applying the package *ConsensusClusterPlus,*^88^ using 1000 iterations with 90% of sampling, “hclust” as method and “ward.D2” as inner and final linkage. As an input, the median-centered expression of the 100 most variable glycosylation genes was used. To find consensus clusters between the different datasets, we used cluster of clusters using *ConsensusClusterPlus* as reported before.^94^ For characterization of the clusters, we used the dataset from Klijn et al. to perform Gene Set Enrichment Analysis (GSEA)^18^ as implemented in the javaGSEA Desktop Application and visualized in Cytoscape using the EnrichmentMap app.^89^ The *limma*^90^ package was used for the differential gene expression analysis between the different clusters.

The method *ssGSEA* of the *GSVA* package to generate scores for selected glycosylation-related pathways.^93^ An EMT score was calculated as described for the scRNA-Seq analysis. Spearman correlation coefficients were calculated using the function *corr.test* of the package *psych*, using FDR to adjust p-value for multiple tests. Only cell lines derived from epithelial tumors were used (Breast, Colorectal, Ovarian, Pancreatic and Stomach cancer).

### Quantification and statistical analysis

All statistical details, such as the specific tests employed and the p values, can be located in the results, methods, and/or figure legends.

## Supplemental information

Document S1. Figures S1–S13 and Table S3

Table S1. List of Glycosylation-related genes used in this article, related to Figure 1

Table S2. List of Glycosylation-related genesets used in this article, related to Figure 1

Table S4. Integrated table of the cell lines included in the studies used in this article, related to Figure 4
