# Supplementary material for: The transcriptional landscape of glycosylation-related genes in cancer
Source: iScience. 2024 Jan 29;27(3):109037. doi: 10.1016/j.isci.2024.109037 (PMC10879703; doi:10.1016/j.isci.2024.109037)
Supplement: Document S1. Figures S1–S13 and Table S3 [file mmc1.pdf]

iScience, Volume *27*

Supplemental information

The transcriptional landscape

# of glycosylation-related genes in cancer

Ernesto Rodriguez, Dimitri V. Lindijer, Sandra J. van Vliet, Juan J. Garcia Vallejo, and Yvette van Kooyk

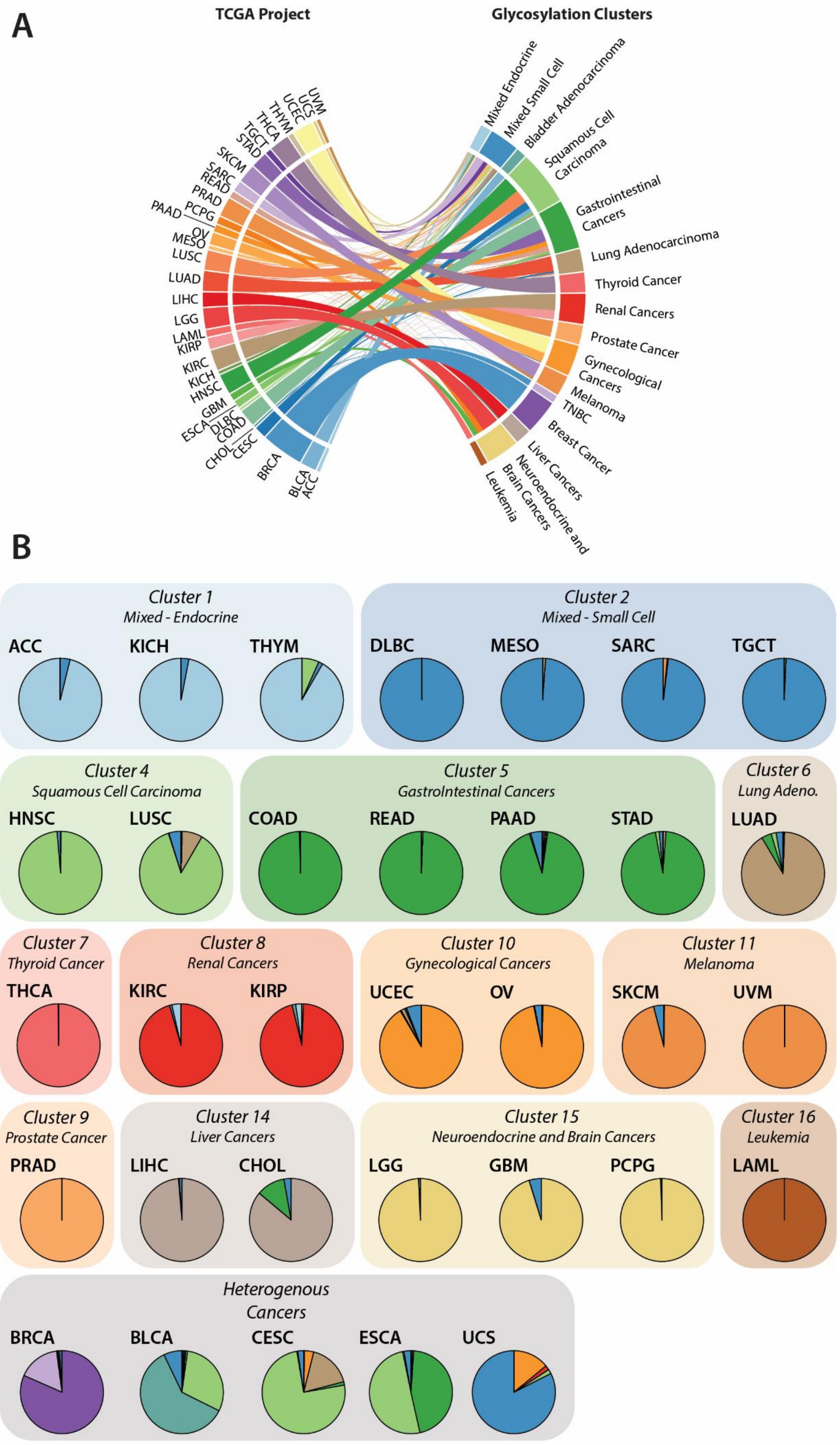

Figure S1, Related to Figure 1. Association of glycosylation clusters with cancer types. A) Chord diagram showing the relation between the different TCGA projects and the glycosylation clusters. B) Pie Charts displaying the quantification of glycosylation clusters in each cancer type. For each TCGA project, the proportion of the different glycosylation clusters was calculated. A cancer type was considered heterogeneous (bottom) when the more abundant glycosylation cluster constituted 85% or less of the samples.

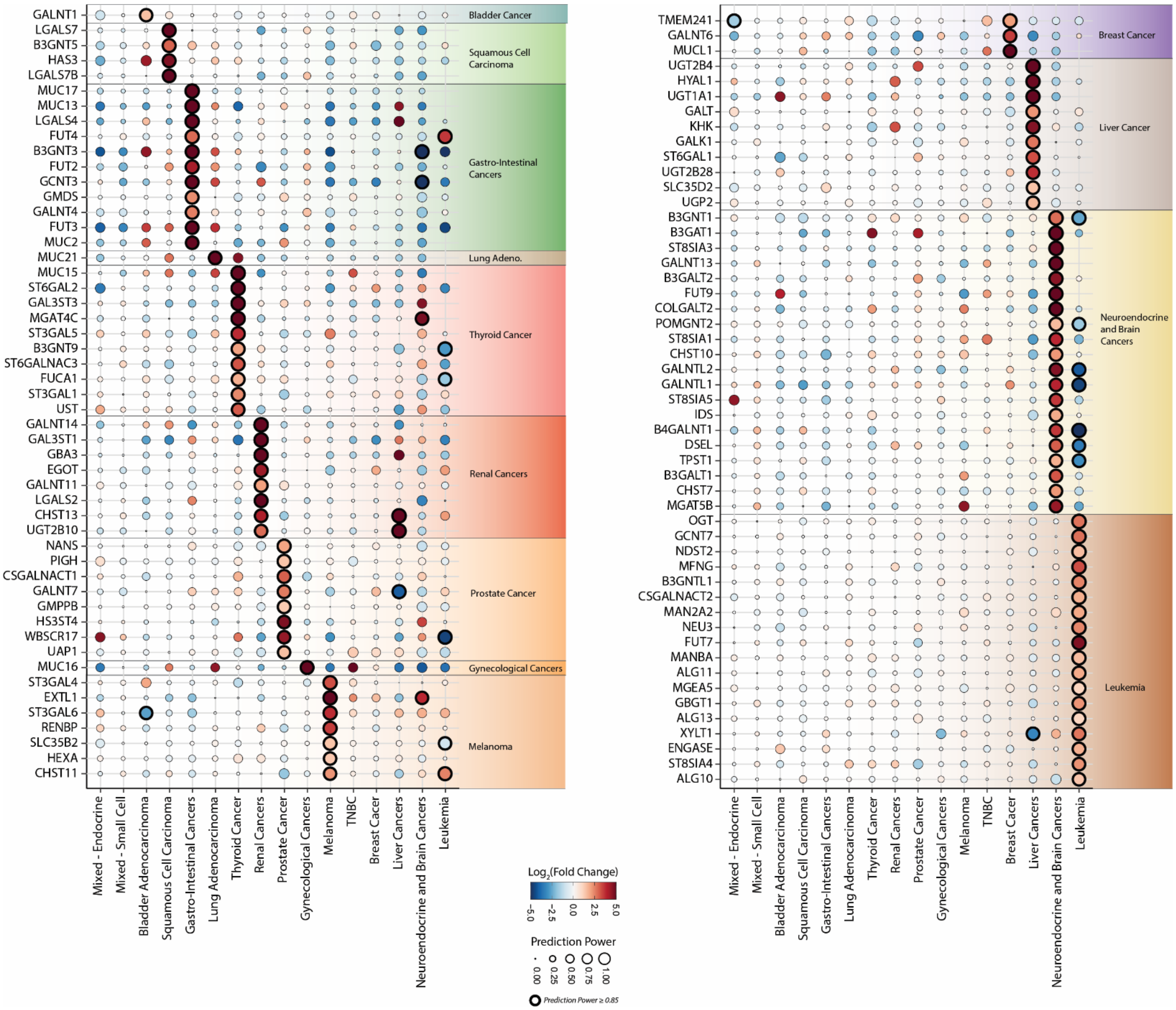

Figure S2, Related to Figure 1. Association of glycosylation-related genes with the different clusters in the TCGA data set. Results of differential expression of glycosylation-related genes using ROC curves. Bold circles indicate results with a prediction power of at least 0.85.

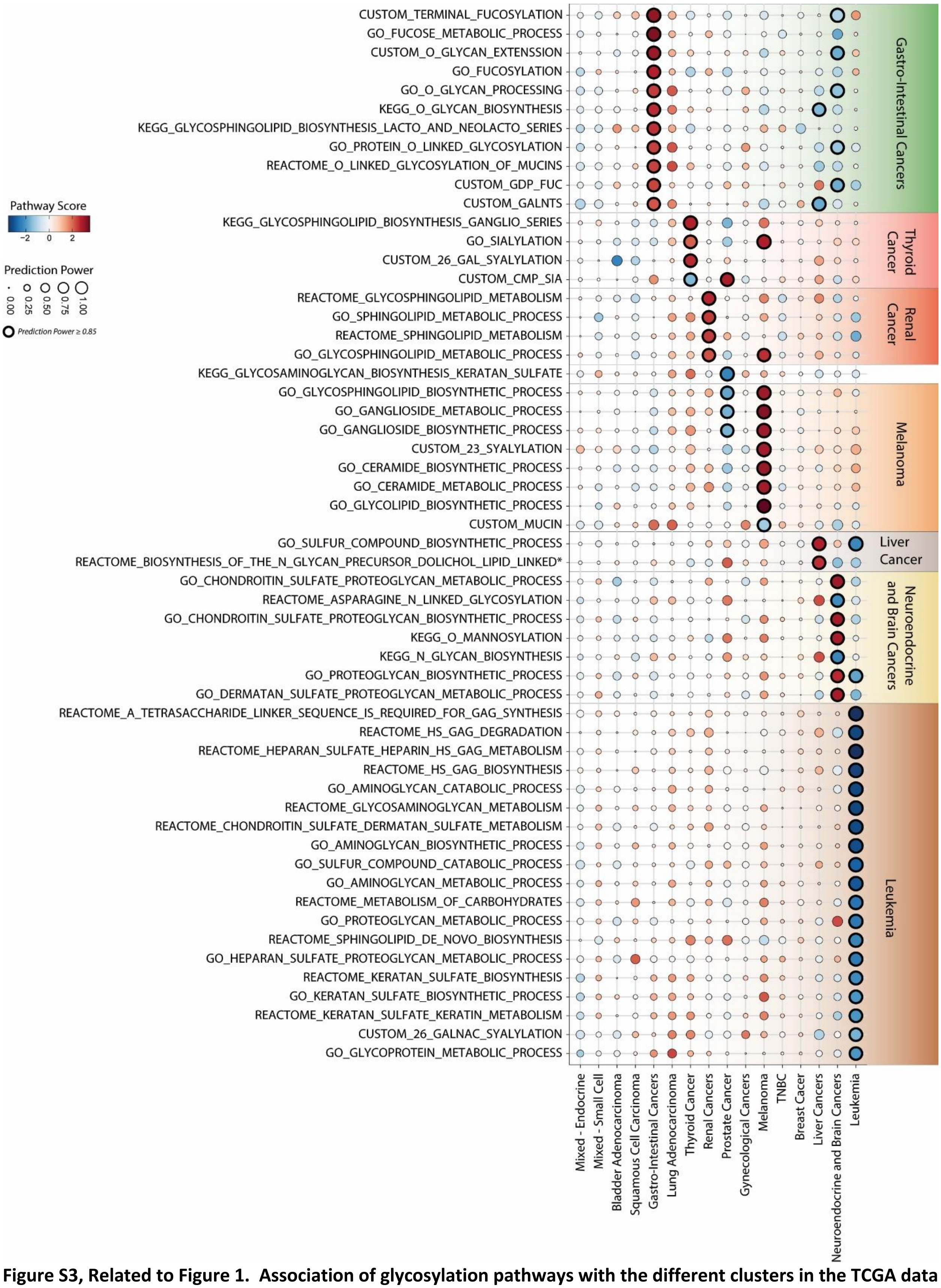

Figure S3, Related to Figure 1. Association of glycosylation pathways with the different clusters in the TCGA data set. Results of differential expression of glycosylation-related pathways using ROC curves. Bold circles indicate results with a prediction power of at least 0.85.

*Full name of gene set: *REACTOME_BIOSYNTHESIS_OF_THE_N_GLYCAN_PRECURSOR_DOLICHOL_LIPID_LINKED_OLIGOSACCHARIDE_LLO_AND_TRANSFER_TO_A_NASCENT_PROTEIN*.

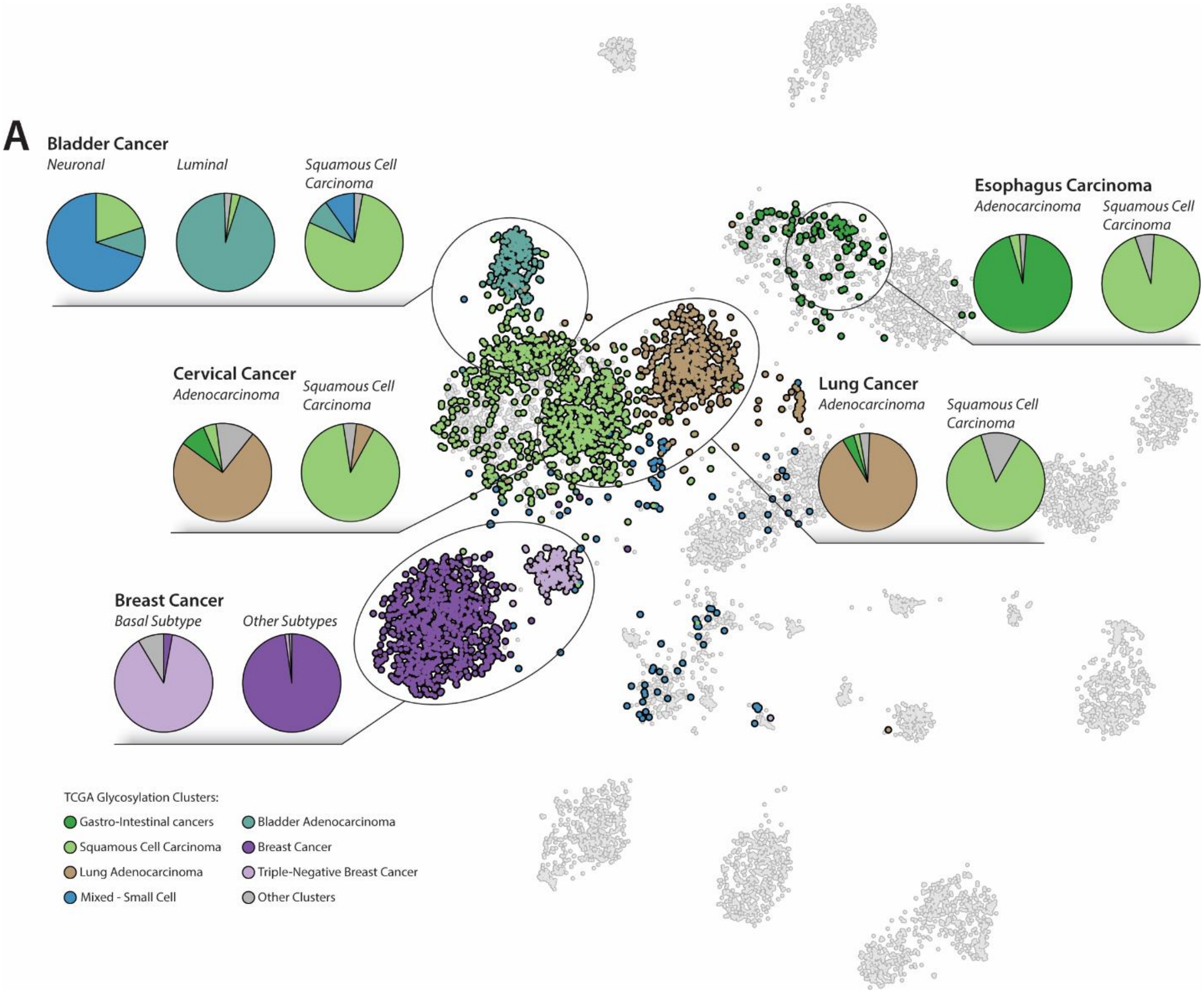

Figure S4, Related to Figure 1. Association of glycosylation-related cluster with different diagnosis or molecular subtypes in heterogenous cancers. For some of the heterogenous cancer defined in Figure S1, we studied the correlation with molecular or histological subtypes. Pie charts represent the proportion of glycosylation clusters present in the indicated cancer subtypes.

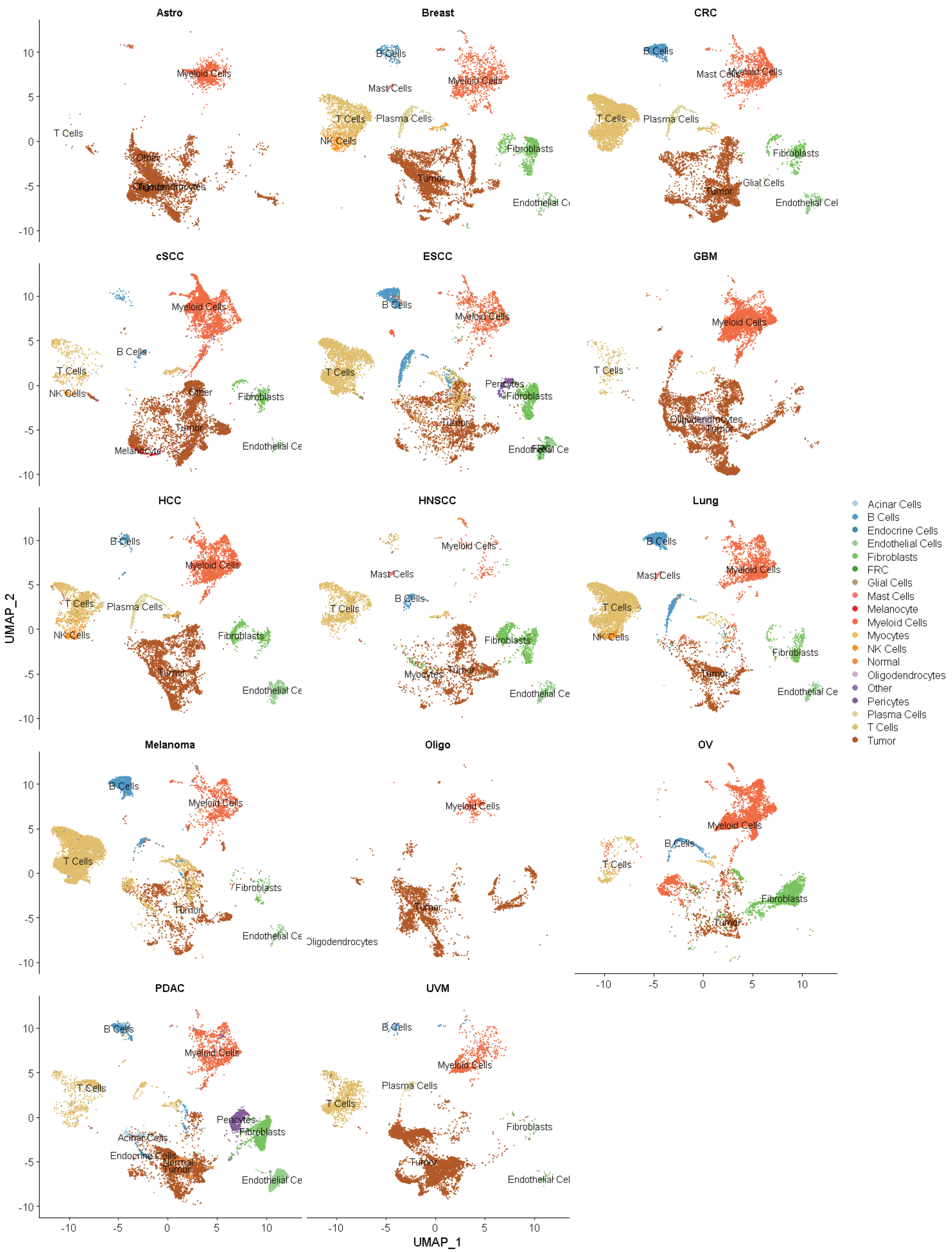

Figure S5, Related to Figure 2. UMAP plot of the integrated single cell RNA-seq dataset displaying the clusters obtained in the independent analysis of each dataset.

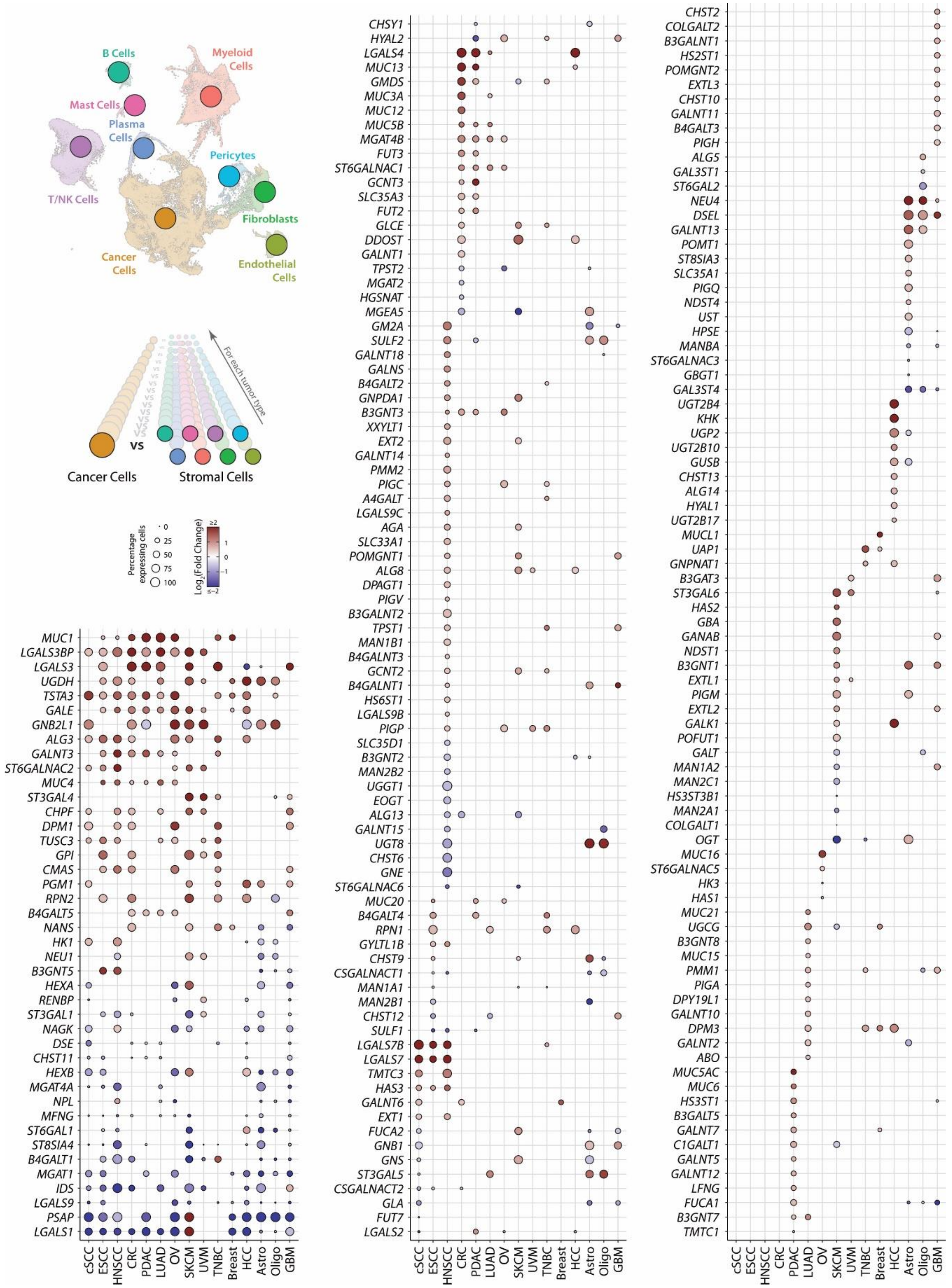

Figure S6, Related to Figure 2. Analysis of the contribution of the cancer and stromal compartment to the expression of GRGs in tumor tissue. In the integrated data set, we performed differential expression of GRGs in each cell type against all the others in every tumor type individually, using a modified version of the function *FindConservedMarkers* of the package *Seruat*.

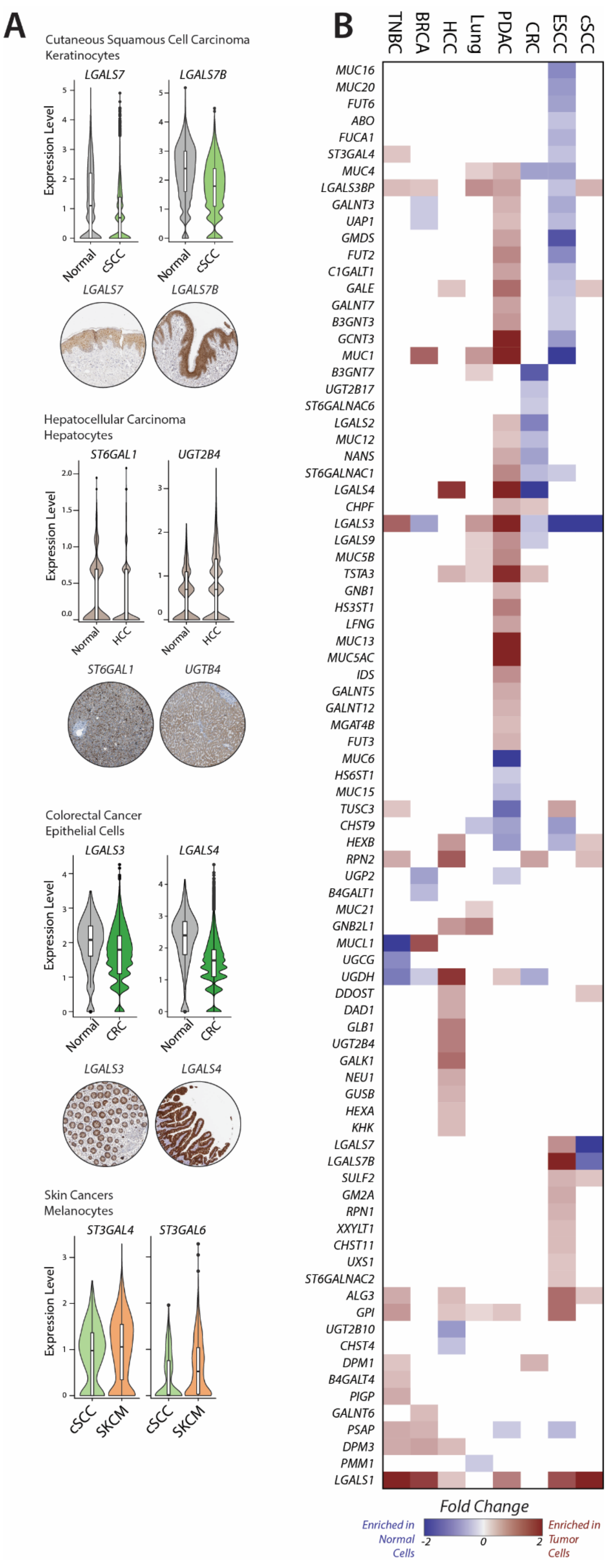

Figure S7, Related to Figure 2. Differential expression of glycosylation-related between normal and tumor cells in scRNA-Seq. A) Top: Expression of specific GRGs in normal and tumor cells in different tumor types. Bottom: Immunohistochemistry staining of different glycosylation-related proteins in normal tissue. Images obtained from the Human Protein Atlas, available from v21.proteinatlas.org, using the corresponding gene names displayed. B) For each dataset, we used the function *FindMarkers* of the *Seurat* package to analyze the differential expression of glycosylation-related genes. The following cell types were used for the analysis between normal and tumor tissue: epithelial cells in in Pancreatic, Colorectal, Lung, Esophagus and Breast cancer, keratinocytes in cutaneous SCC and Hepatocytes in Liver cancer. In violin plot: data presented as boxplot indicate the median, 25th and 75th percentiles (hinges) and whiskers represent 1.5 times the interquantile range.

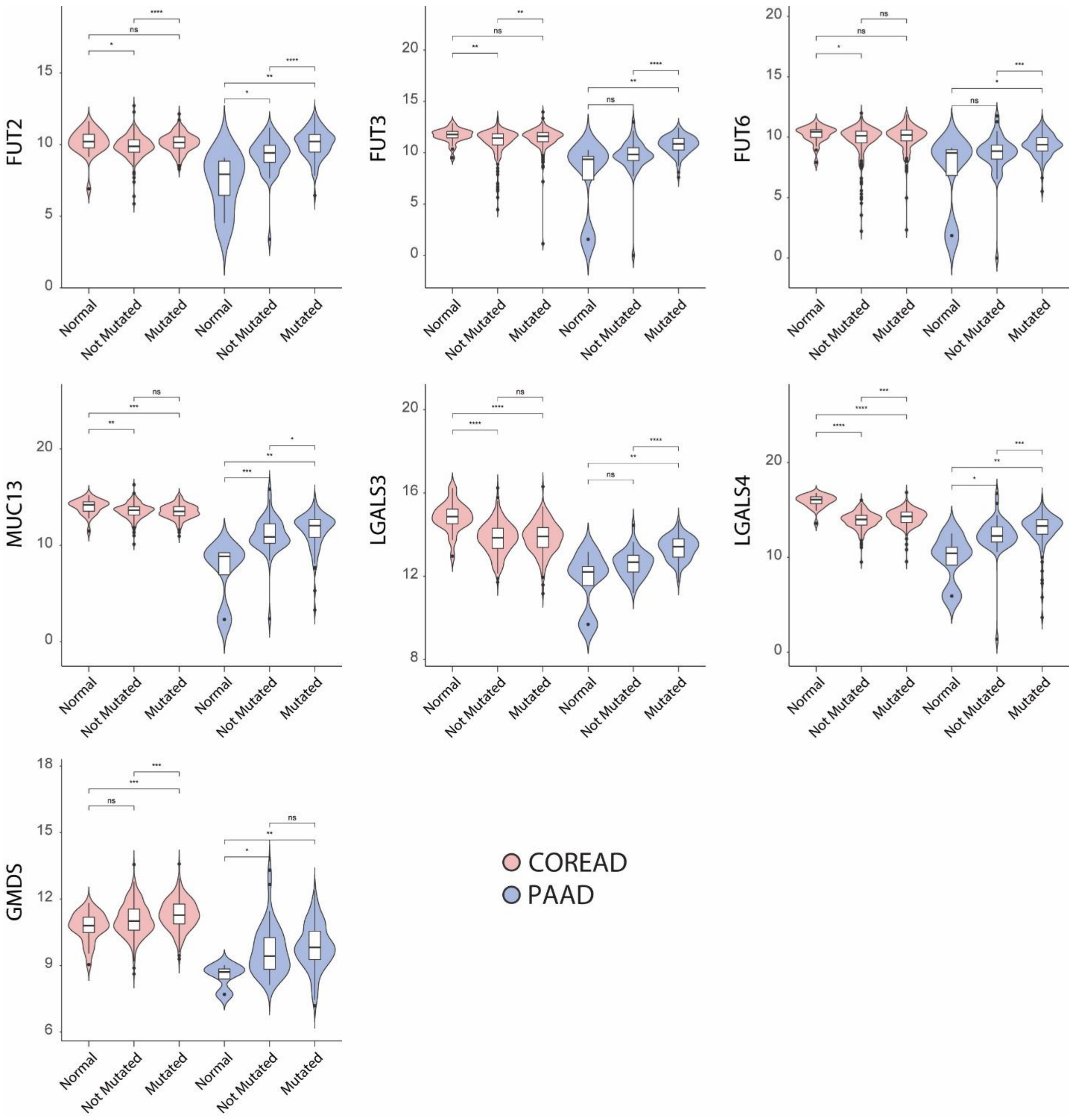

Figure S8, Related to Figure 2. Expression of GRGs associated with gastrointestinal cancers in normal and tumor tissue with or without KRAS mutations. Samples corresponding to the Gastro-Intestinal cluster were used. In COREAD: Normal (n = 26); Not Mutated (n = 256); Mutated (n = 200). In PAAD: Normal (n=4); Not Mutated (n = 43); mutated (n = 109). Statistics: Wilcoxon test (*p ≤ 0.05; **p ≤ 0.01; ***p ≤ 0.001; ****p ≤ 0.0001). In violin plot: data presented as boxplot indicate the median, 25th and 75th percentiles (hinges) and whiskers represent 1.5 times the interquantile range.

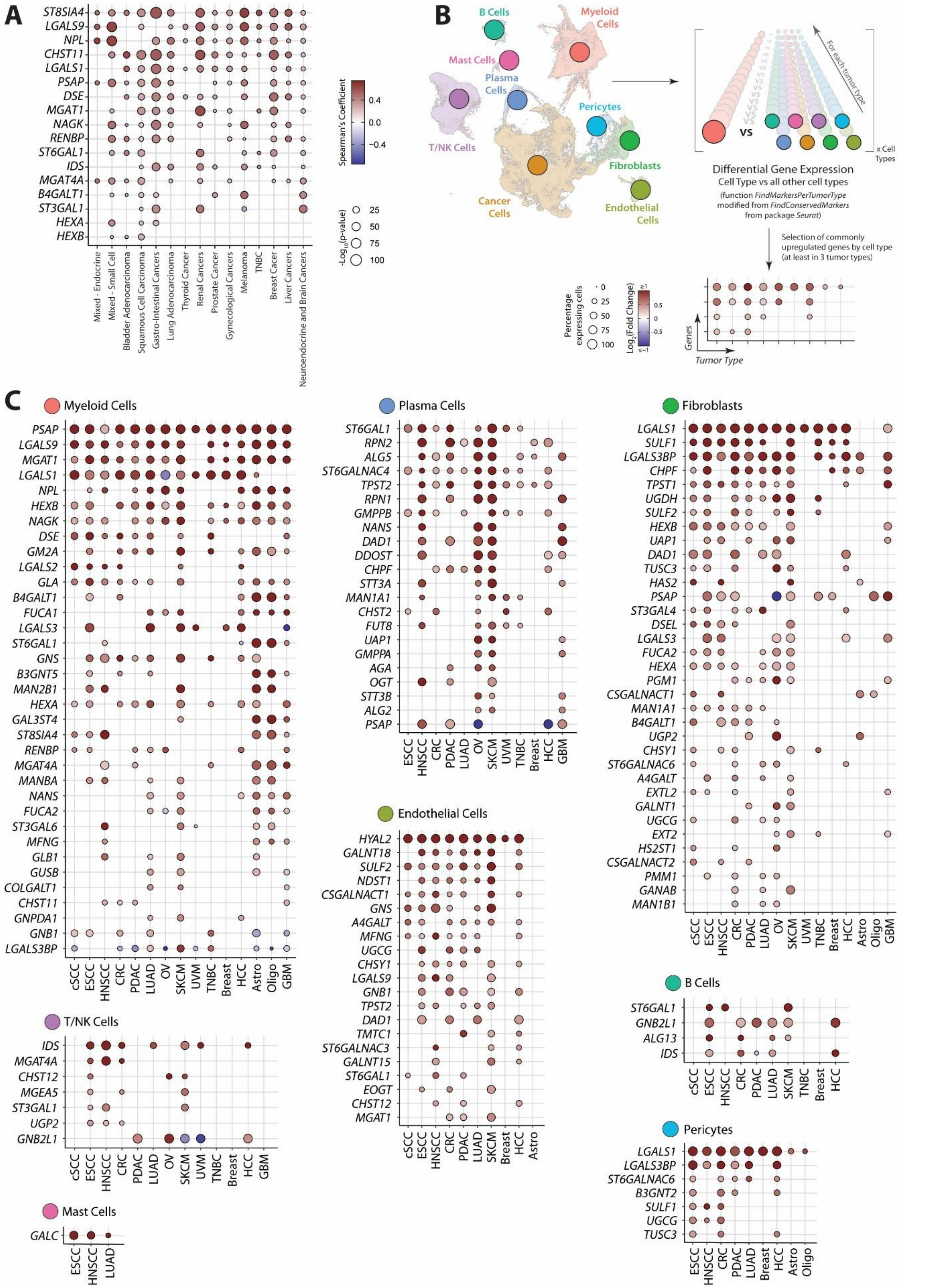

Figure S9, Related to Figure 3. Analysis of the contribution of stromal cells to the expression of GRGs in tumor tissue. A) Correlation between the stromal fraction in the TCGA dataset and the expression stromal-associated GRGs (identified in scRNA-Seq data, Figure S5). B) In the integrated data set, we performed differential expression of GRGs in each cell type against all the others in every tumor type individually, using a modified version of the function *FindConservedMarkers* of the package *Seruat*. C) Dot plot showing the expression of cell specific GRGs.

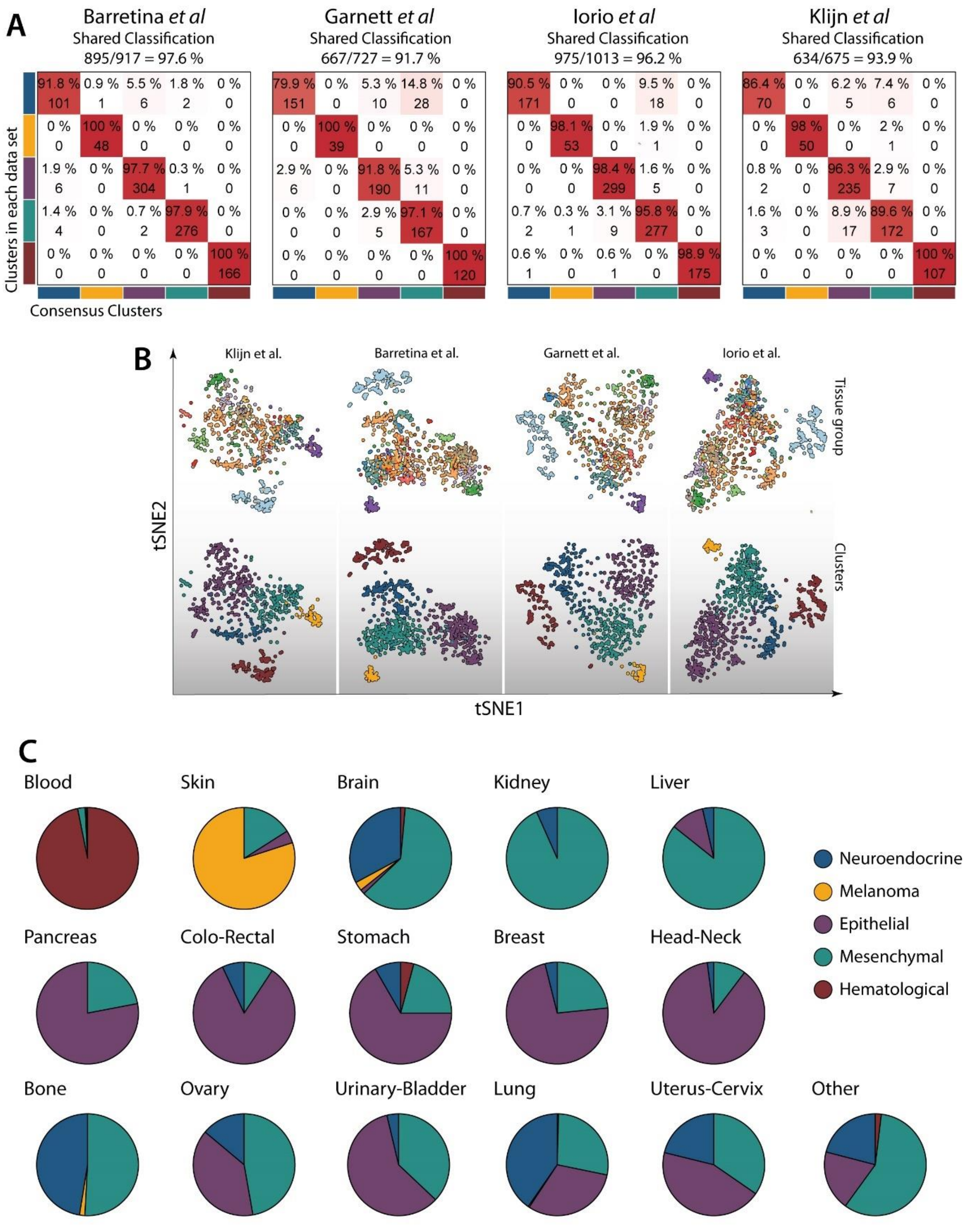

Figure S10, Related to Figure 4. Characterization of different Glycosylation-related clusters in cancer cell lines. A) Confusion matrix of the classification of glycosylation clusters in the individual data sets compared with the consensus clusters. The percentage of the same classification between the two clustering are stated at the top of each matrix. B) TSNE plot of cell lines in the different data sets colored by organ of origin and consensus cluster. C) Pie charts showing the quantification of consensus clusters for each organ of origin of the different cell lines.

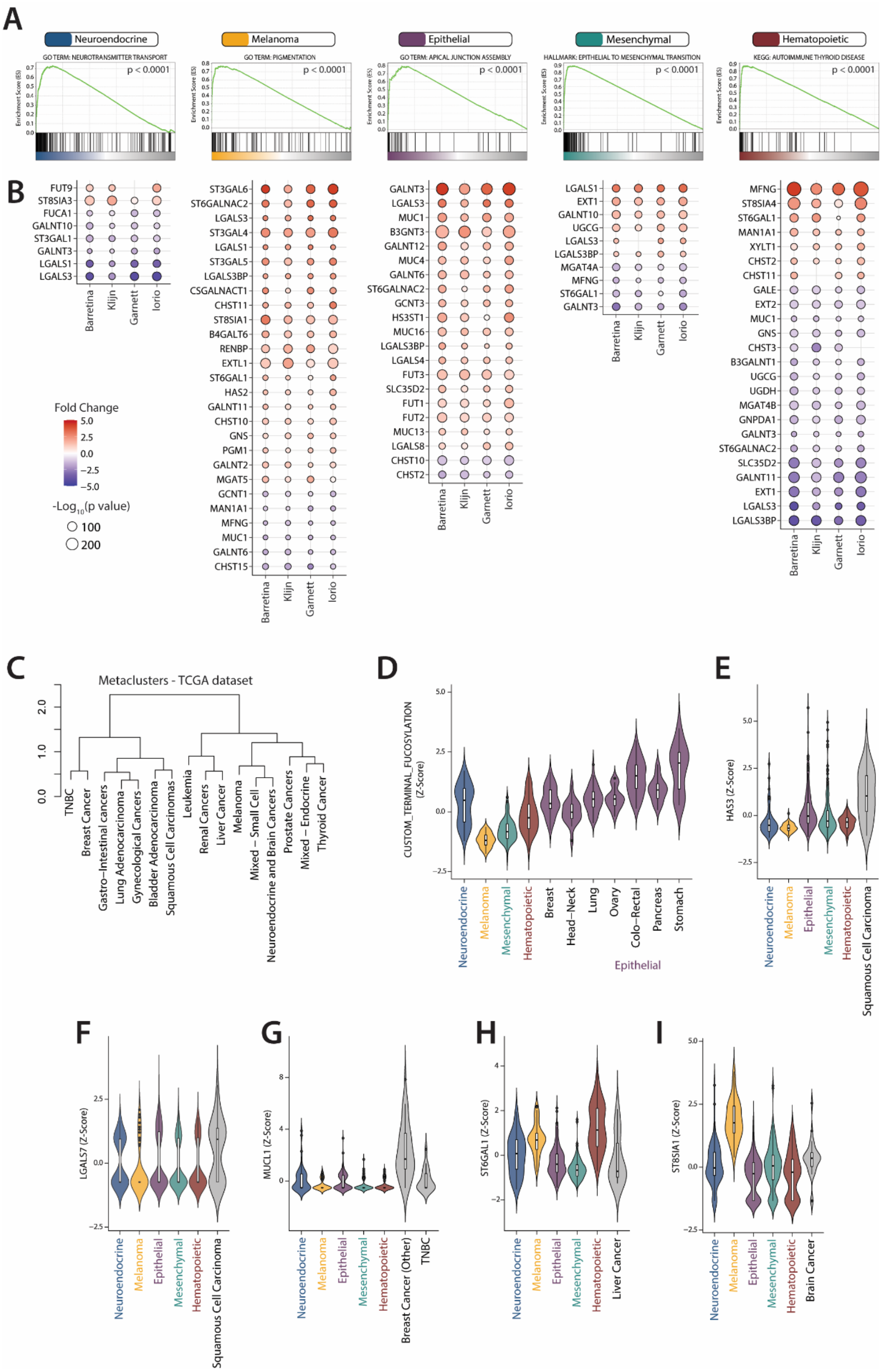

Figure S11, Related to Figure 4. Characterization of different Glycosylation-related clusters in cancer cell lines. A) GSEA plots of selected gene sets enriched in each glycosylation cluster. B) Dot plot of the differential expression of glycosylation-related in glycosylation clusters in each data set. C) Dendrogram displaying the results of the metacluster obtained from the hierarchical clustering of tissue samples. Expression of the geneset "CUSTOM_TERMINAL_FUCOSYLATION" (D) or the genes HAS3 (E), LGALS7 (F), MUCL1 (G), ST6GAL1 (H) and ST8SIA1 (I) in the different subtypes of cell lines. In violin plot: data presented as boxplot indicate the median, 25th and 75th percentiles (hinges) and whiskers represent 1.5 times the interquantile range.

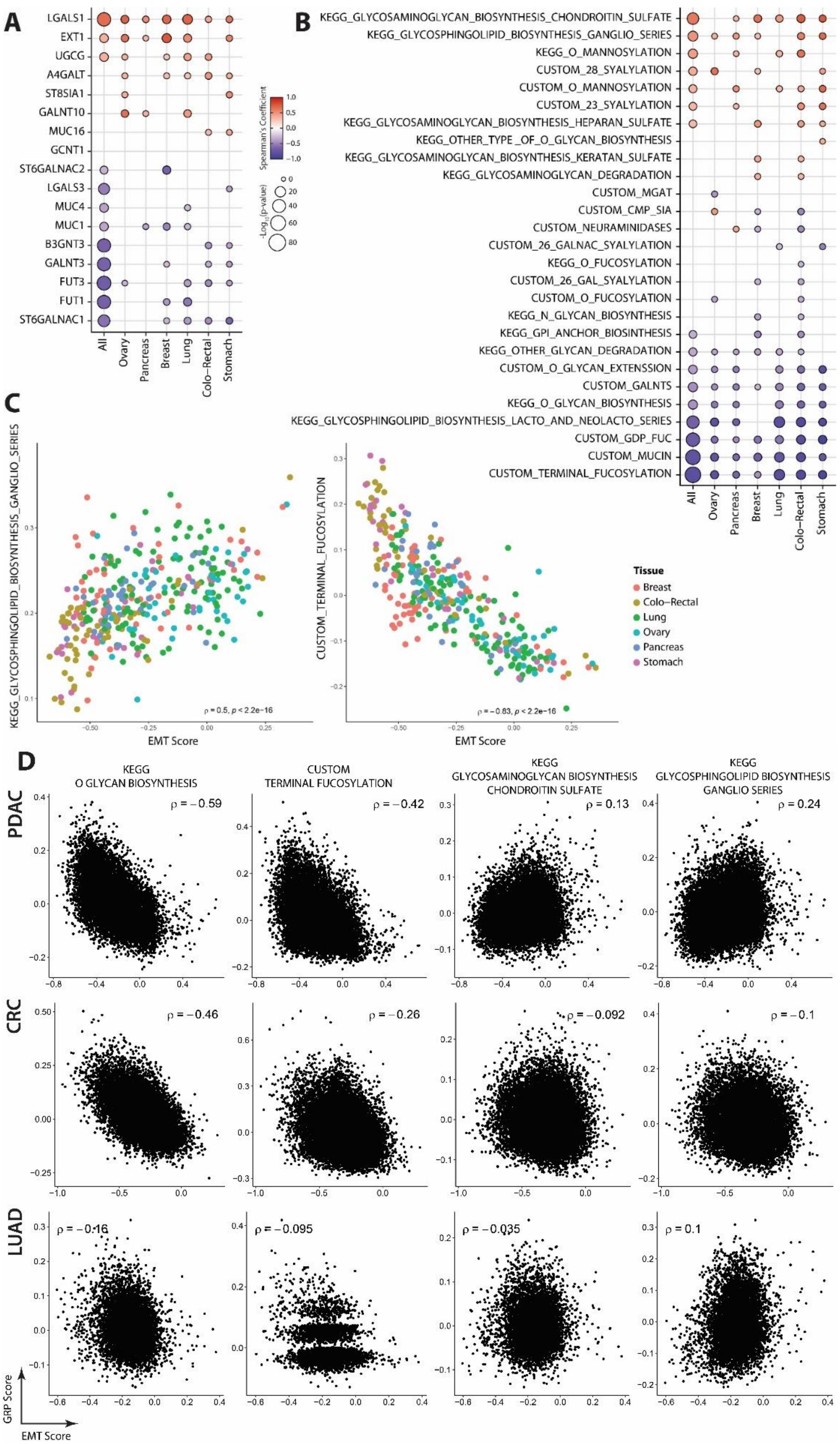

Figure S12, Related to Figure 2. Correlation of glycosylation signatures with Epithelial to Mesenchymal transition. Spearman **corre**lation between an EMT Score and glycosylation-related genes (A) and pathways (B) in cell lines from Ovary, Pancreas, Breast, Lung, Colo-Rectal and Stomach cancers. C) Scatter plot visualizing the correlation of selected glycosylation-related pathways with EMT Score in cancer cell lines. D) Correlation of selected glycosylation-related pathways in scRNA-Seq data of different tumor types.

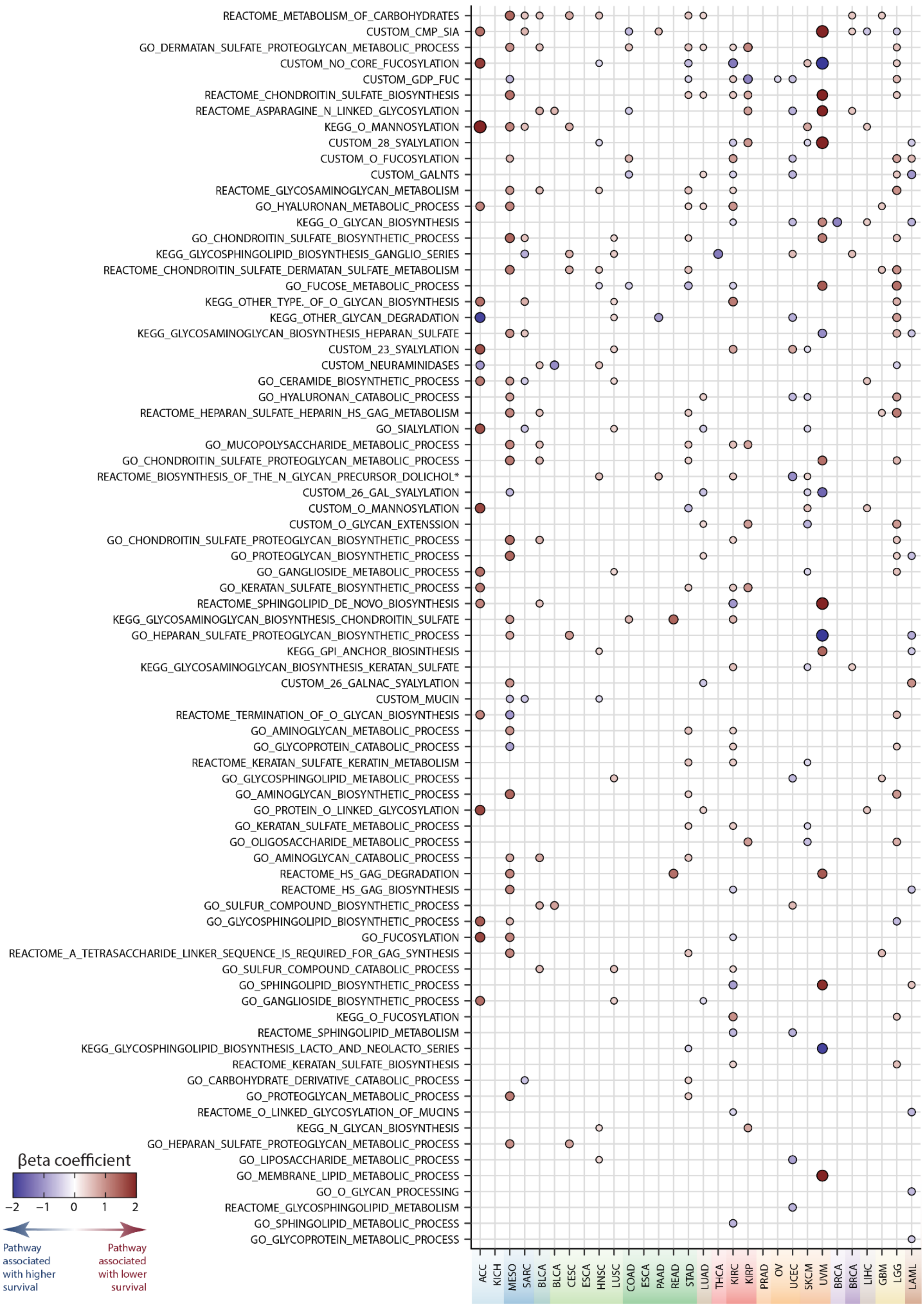

Figure S13, Related to Figure 5. Association of glycosylation pathways with survival in different cancer types.

Table S3, Related to Figure 1 and 2. Transcriptomic datasets obtaines from patients samples used in this manuscript.

TCGA dataset

Project	Description	# Samples	
		Tumor	Normal
ACC	Adrenocortical carcinoma	79	0
BLCA	Bladder Urothelial Carcinoma	408	19
BRCA	Breast invasive carcinoma	1105	114
CESC	Cervical squamous cell carcinoma and endocervical adenocarcinoma	307	3
CHOL	Cholangiocarcinoma	36	9
COAD	Colon adenocarcinoma	454	41
DLBC	Lymphoid Neoplasm Diffuse Large B-cell Lymphoma	48	0
ESCA	Esophageal carcinoma	185	11
GBM	Glioblastoma multiforme	169	5
HNSC	Head and Neck squamous cell carcinoma	522	44
KICH	Kidney Chromophobe	66	25
KIRC	Kidney renal clear cell carcinoma	534	72
KIRP	Kidney renal papillary cell carcinoma	291	32
LAML	Acute Myeloid Leukemia	173	0
LGG	Brain Lower Grade Glioma	534	0
LIHC	Liver hepatocellular carcinoma	374	50
LUAD	Lung adenocarcinoma	517	59
LUSC	Lung squamous cell carcinoma	502	51
MESO	Mesothelioma	87	0
OV	Ovarian serous cystadenocarcinoma	309	0
PAAD	Pancreatic adenocarcinoma	179	4
PCPG	Pheochromocytoma and Paraganglioma	184	3
PRAD	Prostate adenocarcinoma	498	52
READ	Rectum adenocarcinoma	161	10
SARC	Sarcoma	263	2
SKCM	Skin Cutaneous Melanoma	473	1
STAD	Stomach adenocarcinoma	415	35
TGCT	Testicular Germ Cell Tumors	156	0
THCA	Thyroid carcinoma	513	59
THYM	Thymoma	120	2
UCEC	Uterine Corpus Endometrial Carcinoma	533	34
UCS	Uterine Carcinosarcoma	57	0
UVM	Uveal Melanoma	80	0

scRNA-Seq datasets

Project	Description	# Samples		Accession number
		Tumor	Normal	
ESCC	Esophageal Squamous cell carcinoma	60	4	GSE160269
cSCC	cutaneous Squamous cell carcinoma	10	0	GSE144236
COREAD	Colorectal Cancer	23	10	GSE132465
BRCA	Breast Cancer	39	13	GSE161529
SKCM*	Melanoma	48	0	GSE120575
SKCM	Melanoma	31	0	GSE115978
UVM	Uveal melanoma	11	0	GSE139829
OV	Ovarian cancer	11	0	GSE146026
GBM	Glioblastoma	9	0	GSE131928
LUAD	Lung adenocarcinoma	11	11	GSE131907
HCC2	Hepatocellular carcinoma	10	8	GSE149614
HNSCC	Head and neck Squamous cell carcinoma	18	0	GSE103322
LGG	Low grade glioma – Oligodendroglioma	6	0	GSE70630
LGG	Low grade glioma – Astrocytoma	10	0	GSE89567
PAAD	Pancreatic Ductal Adenocarcinoma	24	11	PRJCA00106317

* Only CD45+ Cells
